# Short-term starvation inhibits CD36 N-glycosylation and downregulates USP7 UFMylation to alleviate RBPJ-maintained T cell exhaustion in liver cancer

**DOI:** 10.7150/thno.110567

**Published:** 2025-04-28

**Authors:** Banglun Pan, Siyan Chen, Hao Wu, Xiaoxia Zhang, Zhu Zhang, Dongjie Ye, Yuxin Yao, Yue Luo, Xinyu Zhang, Xiaoqian Wang, Nanhong Tang

**Affiliations:** 1 Department of Hepatobiliary Surgery and Fujian Institute of Hepatobiliary Surgery, Fujian Medical University Union Hospital, Fuzhou 350001, China.; 2 Department of Laboratory Medicine, Fujian Medical University Union Hospital, Fuzhou 350001, China.; 3 Cancer Center of Fujian Medical University, Fujian Medical University Union Hospital, Fuzhou 350001, China.; 4 Key Laboratory of Clinical Laboratory Technology for Precision Medicine (Fujian Medical University), Fujian Province University; Fuzhou 350122, China.; 5 Key Laboratory of Ministry of Education for Gastrointestinal Cancer, Fujian Medical University, Fuzhou 350122, China.

**Keywords:** STS, USP7, RBPJ, UFMylation, ubiquitination, T cell exhaustion, HCC

## Abstract

**Rationale:** Short-term starvation (STS) has been shown to enhance the sensitivity of tumors to chemotherapy while concurrently safeguarding normal cells from its detrimental side effects. Nonetheless, the extent to which STS relies on the anti-tumor immune response to impede the progression of hepatocellular carcinoma (HCC) remains uncertain.

**Methods:** In this study, we employed mass cytometry, flow cytometry, immunoprecipitation, immunoblotting, CUT&Tag, RT-qPCR, and DNA pull-down assays to evaluate the relationship between STS and T-cell antitumor immunity in HCC.

**Results:** We demonstrated that STS alleviated T cell exhaustion in HCC. This study elucidated the mechanism by which STS blocked CD36 N-glycosylation, leading to the upregulation of AMPK phosphorylation and the downregulation of USP7 UFMylation, thus enhancing ubiquitination and destabilized USP7. Consequently, diminished USP7 levels facilitated the ubiquitination and subsequent degradation of RBPJ, thereby inhibiting T cell exhaustion through the IRF4/TNFRSF1B axis. From a therapeutic standpoint, STS not only suppressed the growth of patient-derived orthotopic xenografts but also enhanced their sensitivity to immunotherapy.

**Conclusions:** These findings uncovered a novel mechanism by which N-glycosylation participated in UFMylation/ubiquitination to regulate T cell exhaustion, and we underscored the potential of targeting USP7 and RBPJ in anti-tumor immunotherapy strategies.

## Introduction

Short-term starvation (STS) closely relates to immune system balance by regulating chemokine expression and immune cell activity 1. It boosts infiltration of beige fat cells and M2 macrophages in white adipose tissue 1, controls immune cell circulation and colonization 2, 3, and activates AMPK/PPARα signaling to raise CCL2 levels, inhibiting monocyte movement from bone marrow to the periphery 2. Additionally, fasting increases CXCR4 expression on monocytes through corticosterone release, prompting their migration back to the bone marrow 3. Insufficient energy affects immune response via autophagy, with fasting-induced autophagy supplying antigens to MHC Class II molecules, crucial for dendritic cells to activate T cells 4. However, the role of STS in T cell exhaustion and anti-tumor responses is unclear.

UFMylation, a new ubiquitin-like modification, involves a three-enzyme process: activation enzyme E1 (UBA5), binding enzyme E2 (UFC1), and ligase E3 (UFL1) 5. However, its modified substrate spectrum, the regulatory mechanism and the biological process are still unclear, and only a few substrates have been identified 5. While UFMylation is known to regulate embryonic development 6, endoplasmic reticulum homeostasis 7, DNA damage response 8, and interferon response 9, its impact on immune cells in the tumor microenvironment (TME) is not well understood. Given the importance of T cells in tumor control and immunotherapy, further investigation into UFMylation's role in T-cell-mediated anti-tumor immunity is warranted.

In this study, we found that STS decreased USP7 UFMylation by blocking CD36 N-glycosylation, hindering CD36 membrane localization, increasing AMPK phosphorylation, thus reducing USP7 UFMylation. Reduced levels of this modification promoted USP7 ubiquitination and decreased its stability. As a deubiquitinase, USP7 inhibited RBPJ ubiquitination, enhancing its expression. RBPJ then bound to the promoters of exhaustion gene *Irf4* 10 or *Tnfrsf1b* 11, leading to T cell exhaustion. Therefore, STS connected glucose metabolism and T cell exhaustion via three protein post-translational modifications (PTMs), highlighting its significant role in the regulation of T cell-mediated immunotherapy.

## Methods

### Study design

Details regarding the number of biological replicates, the statistical methods employed, and the *P* values were provided in the figure legends. Sample sizes were not predetermined using statistical methods; instead, they were estimated based on preliminary experiments. All *in vitro* cellular functional experiments were conducted a minimum of three times, with no exclusion from outliers or other data points from our analyses. Animal experiments were conducted at least five times, with mice being randomly assigned to treatment groups by cage. Investigators were blinded to group assignments during the experiments and outcome assessments. Given the post hoc nature of the study, the analyses presented should be considered exploratory.

### T cell treatment strategies

Various mouse HCC models were constructed (**Mouse model**). Following the sacrifice of mice, primary HCC tissues were dissociated into single cell suspensions (**Isolation of tumor tissue**). Subsequently, live T cells were isolated through flow cytometry sorting (**Flow cytometry analysis or sorting**). Cell transfection, immunoblotting, immunoprecipitation and RT-qPCR were then employed.

### Construction

The target genes were PCR-amplified from cDNA, cloned into the pDNA3.1 vector (V79020, Addgene, MA, USA), and tagged with 6×His, HA, or Myc at the C-terminus.

### Cell lines

HEK293T (300189, Cytion, Germany) and Hep-53.4 (400200, Cytion) cells were grown in DMEM (10569010, Gibco, NY, USA) with 10% FBS (10099158, Gibco) and 1% Pen-Strep (HY-K1006, MedChemExpress, NJ, USA), while mouse primary T cells were cultured in T Cell Expansion SFM (A1048501, Gibco) with the same supplements. To ensure the proliferation of T cells, Dynabeads Mouse T-Activator CD3/CD28 (11456D, Gibco) was used to activate them. T cells were cultured in RPMI 1640 medium (11879020, Gibco) with the same supplements for glucose starvation. All cultures were maintained at 37°C with 5% CO_2_. T cells were treated with A-769662 (3 μM, HY-50662, MedChemExpress), Cycloheximide (500 nM, HY-12320, MedChemExpress), Dorsomophine (10 μM, S7840, Selleck, TX, USA), MG132 (40 μM, HY-13259, MedChemExpress), PNGase F (500U, HY-P2929, MedChemExpress), Swainsonine (40 μM, HY-N6722, MedChemExpress), and Tunicamycin (2 μg/mL, HY-A0098, MedChemExpress) for 48 h, respectively.

### Mouse model

All animal experiments followed protocols approved by Fujian Medical University's Ethics Committee (IACUC FJMU 2023-Y-0841). Mice were kept in ventilated cages in a pathogen-free environment at room temperature (RT) and 50%-60% humidity. Six mice were randomly assigned per group. Specific operation strategy:

**1)** Construction of subcutaneous tumor model: Hep-53.4 cells (5 × 10^6^) were suspended in 100 μL DPBS (14190144, Gibco) and injected subcutaneously into 10-week-old female C57BL/6J mice (~20 g, GemPharmatech, China). Tumor formation was observed when the control group's average tumor diameter reached at least 2 mm. When a mouse's tumor reached 2 cm in diameter, it was euthanized with CO_2_, and the tumor was removed, photographed, and weighed. Tumor volume was calculated using the formula: (long axis × wide axis²) × 0.5.

**2)** Construction of primary HCC model: Briefly, a single dose of DEN (25 mg/kg, HY-N7434, MedChemExpress) was injected into 15-day-old female C57BL/6J mice intraperitoneally (*i.p.*) to initiate tumor formation. At 4 weeks of age, CCl_4_ (0.5 mL/kg, HY-Y0298, MedChemExpress) was injected *i. p*. Twice a week with CCl_4_ for an additional 12 weeks. When HCC was formed in the liver, and confirmation was obtained by bimanual palpation and dissection. Anesthesia and euthanasia during the experiment: If an animal becomes listless and loses its appetite, it should be euthanized with CO_2_.

**3)** Construction of patient-derived orthotopic xenograft (PDOX) model: The study involving patients was approved by the Ethics Committee of Fujian Medical University Union Hospital (approval number [2023]173) and performed in accordance with the Helsinki Declaration and government policies. Briefly, mechanically minced pieces of fresh patient HCC tissue were plated on bottles at 37°C in Human Liver Organoid Culture Medium (abs9529, Absin, China) for up to 2 weeks. Organoids with a diameter of 300 µm were made with sterile DPBS at a concentration of 2×10^7^/mL, 1 × 10^6^ cells per mouse, and an injection volume of 50 μL. huHSC-NCG mice (GemPharmatech) were anesthetized with 0.8% Pentobarbital Sodium (60 mg/kg, P3761, Merck, Germany) by *i. p*. After anesthesia, the mice were fixed on the operating board, and the surgical site was disinfected. A longitudinal incision was made about 1 cm below the xiphoid process of mice to open the abdominal cavity, and the left lobe of the liver was gently exposed with a sterile cotton swab. Fix the liver lobe with a cotton swab, insert a 1 mL micro-syringe along the liver surface at 15-30°, penetrate the liver of about 0.5 cm, inject the cell suspension slowly, withdraw the needle slowly, and use a sterile cotton swab to lightly press the injection site to stop bleeding. The liver was then carefully placed back into the abdominal cavity, the abdomen was sutured layer by layer, and the incision was sterilized. Place the mice on an electric blanket until the mice wake up, then return them to their cages and observe the changes in the mice's vital signs and body weight. Mice (passage 1) were maintained and sacrificed when behavioral abnormalities (collapse, hyperactivity) and weight loss occurred. Organoids (passage 1) were further prepared from minced xenograft livers in the same manner as patient tissues and implanted for several generations. The PDOX model was established at passage 3, when tumor phenotype tended to stabilize.

**4)** Construction of *Usp7*- or *Rbpj*-conditional knockout (cKO) mice: To create a *Usp7*-cKO mouse model (T051806, GemPharmatech), Exon 2-Exon5 of *Usp7*-208 transcript were targeted as the flox region. For a *Rbpj*-cKO mouse model (NM-CKO-2101541, Shanghai Model Organisms Center, China), Exon 6-Exon7 were selected. Knocking out these regions would induce the frameshift mutation, leading to premature translation termination and protein mutations. Fertilized eggs were implanted into female C57BL/6J mice to produce positive F0 mice. To achieve T cell-specific deletion, floxed *Usp7* or *Rbpj* mice were bred with *Cd4-Cre* transgenic mice (T004818, GemPharmatech). For animal studies with *Usp7*^flox/flox^*Cd4-Cre* (*Usp7*-cKO) or *Rbpj*^flox/flox^*Cd4-Cre* (*Rbpj*-cKO) mice, littermates with *Usp7*^flox/flox^* or Rbpj*^flox/flox^ as control were used. Deletion of the floxed *Usp7* allele and wild-type (WT) *Usp7* exons was detected by PCR using the following primers: *Usp7*-5'arm (F-GGTCAAAGAGATTCTGAAGTACCCATT, R-AAACTGGCCCACAATCCCTTG), *Usp7*-3'arm (F-GCACTGGTTTTCCAGTCACAGTG, R-CTTCTTCCCAGCAAACTTCTGATC). Deletion of the floxed *Rbpj* allele and WT *Rbpj* exons was detected by PCR using the following primers: *Rbpj*-floxed (F-GAAGGTCGGTTGACACCAGATAGC, R-GCAATCCATCTTGTTCAATGGCC), *Rbpj*-WT (F-GTTCTTAACCTGTTGGTCGGAACC, R-GCTTGAGGCTTGATGTTCTGTATTGC). Primers that produce T cell-specific KO: 5'arm (F-GGGCAGTCTGGTACTTCCAAGCT, R-AGCATGTCTCAGGGTTCAGCCTAG), WT (F-CAGCAAAACCTGGCTGTGGATC, R-ATGAGCCACCATGTGGGTGTC).

**5)** Construction of *Usp7*- or *Rbpj*-conditional knock-in (cKI) mice. Using CRISPR/Cas9 technology, a CAG-tdTomato-polyA expression frame was inserted into the *Rosa26* gene locus by homologous recombination. A homologous recombination vector containing a 5' homology arm, a tdTomato expression frame, and a 3' homology arm was constructed by In-Fusion cloning. Cas9 mRNA, gRNA, and donor vector were microinjected into fertilized eggs of female C57BL/6J mice to obtain mice with loxp sites and mate with *Cd4-Cre* transgenic mice. For animal studies with *Usp7*^flox/flox^*Cd4-Cre* (*Usp7*-cKI) or *Rbpj*^flox/flox^*Cd4-Cre* (*Rbpj*-cKI) mice, littermates with *Usp7*^flox/flox^* or Rbpj*^flox/flox^ as control were used. Primers for *Usp7*- or *Rbpj*-cKI identification: WT primers-used to identify the presence of WT alleles and Cas9 activity (F-CCTCTTCCCTCGTGATCTGC, R-TGGAAAATACTCCGAGGCGG), Insert primers-used to identify whether the Donor inserted into the *Rosa26* locus (F-GGGCAACGTGCTGGTTATTG, R-AAGGGTTCCGGATCAGCTTG). Identification of genotype results: Homozygous: Only Insert primers amplification bands were positive. WT: Only the amplification bands of WT primers were positive. Heterozygote: The amplified bands of both primers were positive.

**6)** Ark313-*Tnfrsf1b*/*Irf4*-overexpression (OE) production and tail vein infusion: Ark313 is a synthetic AAV-6 that exhibits high transduction efficiency in T cells 12. HEK293T cells were co-transfected with 11 μg AAV-6 Packaging System (VPK-406, Cell Biolabs, CA, USA), 8 μg AAV-6 Rep-Cap Plasmid (VPK-426, Cell Biolabs), and 6 μg Ark313 12-*Tnfrsf1b*/*Irf4*-OE plasmid using the AAV-MAX System (A51217, Gibco) for 72 h. Transfected cells were collected in Viral Production Medium (A4817901, Gibco), lysed by three cycles of rapid freeze/thaw, and then incubated with 25 IU/mL Benzonase (E1014, Merck) for 1 h at 37°C. The high-pressure injection via tail vein was performed. Briefly, mice were injected with 2 mL of saline containing Ark313-*Tnfrsf1b*/*Irf4*-OE (5 μg) within 7 s via the tail vein.

**7)** Drug treatment: The drug injection protocol involved administering Abatacept-anti-CTLA4 antibody (10 mg/kg, HY-108829, MedChemExpress), anti-mouse CD3ε antibody (15 mg/kg, A2104, Selleck), anti-mouse IgG antibody (15 mg/kg, A2150, Selleck), Avagacestat (10 mg/kg, HY-50845, MedChemExpress), Camrelizumab-anti-PD1 antibody (3 mg/kg, HY-P9971, MedChemExpress), MK-0752 (100 mg/kg, HY-10974, MedChemExpress), and SB-747651A (5 mg/kg, HY-114038, MedChemExpress) into the tail vein 20 days before the mice were sacrificed, and then every three days for a total of five doses. Equal volume of DMSO (HY-Y0320, MedChemExpress) or normal saline (IN9012, Solarbio, China) was used as the control group.

**8)** STS conditions: Mice underwent complete food deprivation with free access to water for two cycles of 48 h and one cycle of 24 h 13.

### Cell cycle detection

T cells (1 × 10^5^) were fixed overnight with 70% ethanol (100983, Merck). The cells were resuspended in 250 μL of FxCycle™ PI/RNase Staining Solution (F10797, Invitrogen, CA, USA), mixed, and incubated for 20 min at 4°C. Relative light units were detected by BD FACSAria™ III Cell Sorter (BD Biosciences, NJ, USA) within 1 h.

### Cell transfection

For electrotransfection: T cells were suspended in Opti-MEM™ I Reduced Serum Medium (31985062, Gibco) at a concentration of 10^7^ cells/mL. Plasmid DNAs were added into the suspension to achieve a final concentration of 10 mg/mL. The pulses were generated by using a NxT Electroporation System (NEON18SK, Thermo Scientific, CA, USA). After electrotransfection, samples were incubated at 37℃ for 10 min to promote endocytosis. For gene KO, mouse CRISPR target sequence was designed and synthesized: *Ampk* (GAAGATTCGGAGCCTTGACG), *Cd36* (TGTGCAAAACCCAGATGACG), *Hmgcr* (TCATCATCCTGACGATAACG), *Insr* (TTGTTCCGGATGTCCATACC), *Pdl1* (TCCAAAGGACTTGTACGTGG), *Ufl1* (AGAGACGAGCTACATGTCCG), *Ufsp2* (GGGTCGATATCTCCAGCATG), and *Usp7* (GCGCTCGACAGTGAACTGAA).

### CUT&Tag assay

We conducted the CUT&Tag assay using CUT&Tag Assay Kit (77552, Cell Signaling Technology, MA, USA). 100,000 cells were lysed, and the lysate was treated with Con A-coated magnetic beads for 1 h at RT. This was followed by a 2-h incubation with anti-RBPJ antibody (720219, Invitrogen), a 1-h incubation with a secondary antibody (31460, Invitrogen), and a 1-h incubation with a pA/G-Tn5 linker complex. Indexed libraries were pooled based on cell count and sequenced on a NovaSeq 6000 (Illumina, CA, USA) with paired-end 150 bp.

For CUT&Tag data analysis, Fastp 14 was used to trim adaptors and remove low-quality reads, producing high-quality clean reads that were aligned to the mm39 genome. Deeptools 15 calculated and plotted correlation and read distribution heatmaps. MACS3 16 identified peaks, excluding blacklist regions. ChIPSeeker 17 annotated peaks, and Homer identified motifs. MANorm2 18 detected differentially enriched regions between sample groups. ClusterProfiler conducted GO and KEGG enrichment analyses on genes linked to the differentially enriched regions. Enriched peaks were visualized using IGV 19. NewCore BioTechnology (Shanghai, China) provided bioinformatics support.

### Cytometry by time-of-flight (CyTOF)

Antibody labeling: Antibodies were conjugated to isotopically enriched lanthanide metals using the Maxpar X8 Antibody Labeling Kit (NC1648790, Standard BioTools, CA, USA). The labeled antibodies were stored in DPBS supplemented with 1% glycerol (1295731, Merck), 0.05% BSA (AM2616, Invitrogen), and 0.05% sodium azide (S2002, Merck) at 4℃. Antibody used in this study: anti-BTLA (PA5-95592, Invitrogen), anti-CD3E (14-0032-82, Invitrogen), anti-CD4 (14-0041-82, Invitrogen), anti-CD8A (14-0808-82, Invitrogen), anti-CD25 (14-0251-86, Invitrogen), anti-CD27 (14-0271-82, Invitrogen), anti-CD39 (14-0391-82, Invitrogen), anti-CD44 (14-0441-82, Invitrogen), anti-CD45 (14-0451-82, Invitrogen), anti-CD62L (MA1-10262, Invitrogen), anti-CD69 (MA1-207, Invitrogen), anti-CD80 (14-0801-82, Invitrogen), anti-CD95 (14-0951-85, Invitrogen), anti-CD127 (14-1271-82, Invitrogen), anti-CD152 (14-1522-82, Invitrogen), anti-CD160 (14-1601-81, Invitrogen), anti-CD223 (14-2231-82, Invitrogen), anti-CD279 (11-9985-82, Invitrogen), anti-GZMB (PA5-13518, Invitrogen), anti-IFNG (MM700, Invitrogen), anti-IL2 (14-7021-81, Invitrogen), anti-IL6 (M620, Invitrogen), anti-IL21 (PA5-46962, Invitrogen), anti-KI67 (MA5-14520, Invitrogen), anti-KLRG1 (ab25054, Abcam, UK), anti-Perforin (ab261727, Abcam), anti-TBX21 (PA5-109245, Invitrogen), anti-TCF7 (MA5-14965, Invitrogen), anti-TCRβ (14-5961-82, Invitrogen), anti-TCRγδ (14-5711-82, Invitrogen), anti-TIGIT (MA5-48199, Invitrogen), anti-TNFA (PA1-40281, Invitrogen), anti-TOX (A700-212, Invitrogen), anti-VISTA (MA5-48237, Invitrogen), and anti-2B4 (14-2441-82, Invitrogen) antibodies.

Cell staining: Collect single cell suspension, add 1 μL of Cisplatin (201064, Standard BioTools) with a final concentration of 5 μM to distinguish live cells from dead cells, incubate at 37℃ for 5 min, and then add five times the volume of Cell Staining Buffer (201068, Standard BioTools) to terminate the labeling reaction. Centrifuge at 300 × g for 5 min, discard the supernatant, and resuspend the cells in Cell Staining Buffer. To detect cytokine expression, stimulate the cells with Cell Stimulation Cocktail (plus protein transport inhibitors) (00-4975-93, eBioscience, CA, USA) for 6 h. After stimulation, the cells were centrifuged at 300 × g for 5 min and diluted to 1 mL with Cell Staining Buffer to terminate the stimulation. Nonspecific signals were blocked by adding 5 μL Fc Receptor Binding Inhibitor Polyclonal Antibody (14-9161-73, eBioscience), followed by staining with pre-mixed surface antibodies at 4℃ for 30 min. After washing the cells twice with Cell Staining Buffer, the cells were fixed in 200 μL of Fix and Perm Buffer (201067, Standard BioTools) containing 250 nM Intercalator-Ir (201192A, Standard BioTools) at 4℃ overnight. The cells were washed twice with Cell Staining Buffer and stained with premixed intracellular antibodies at 4℃ for 30 min. The cells were washed twice with Nuclear Antigen Staining Buffer (201063, Standard BioTools), resuspended in Cell Acquisition Solution Plus for CyTOF XT (201244, Standard BioTools), and then mixed with 20% EQ Four Element Calibration Beads (201078, Standard BioTools). Data were acquired using a CyTOF XT (Standard BioTools) and saved as fcs files.

Preprocessing of mass cytometry data: Raw data were normalized using the MATLAB version of the Normalizer tool 20. Cells were assigned by manually gating on Event length and DNA (^191^Ir and ^193^Ir) channels, followed by the dead cell discrimination analyzing ^195^Pt expression using a FlowJo (BD Biosciences). Doublets were excluded using Gaussian discrimination channels. Next, data were concatenated and de-barcoded using Boolean gating. The normalized data containing living cells from every individual sample were manually exported from FlowJo and imported into R using the “flowCore” 21 and “flowWorkspaceData” 22. Before automated high-dimensional data analysis, the mass cytometry data were transformed with a cofactor in the range of 5 and 60 using an inverse hyperbolic sine function 23. Living, single cells were exported and imported into R. Before automated high-dimensional data analysis, data were transformed using an inverse hyperbolic sine function with a cofactor in the range of between 300 and 600. Additionally, all data were normalized between 0 and 1 to match the 99-999^th^ percentile of the combined samples in each batch.

Automated subset identification: To identify T cell subsets accurately, we first performed step 1 of FlowSOM clustering on the preprocessed and combined mass cytometry dataset to generate a starting point of 100 nodes 24. The respective k-value was manually chosen (in the range of between 20 and 30); identified subsets were annotated and merged based on a similarity of antigen expression to uphold the biological relevance of the dataset. Manually annotated subsets were used to calculate the relative frequencies of T cell subsets. Heatmaps showed the median expression levels of all markers for each merged subset and were plotted using the “pheatmap”. From mass cytometry datasets, we pre-selected major subsets and performed additional FlowSOM 24 analysis to identify smaller cell subsets. We calculated the median antigen expression among selected cell types of the mass cytometry batch using the “dplyr”. For data visualization, we applied dimensionality reduction techniques. For a complex overview of the immune compartment, we used t-SNE 25. To create a t-SNE of isolated T cells, we pooled equally proportioned 120,000 T cells from the datasets from the CyTOF batch.

### Detection of histone H3 modification

Histone H3 modification levels were detected using EpiQuik Histone H3 Modification Multiplex Assay Kit (P-3100-96, Epigentek, NY, USA). Add 40 ul of sample or standard to the well and incubate at 37°C for 30 min. Wash three times with DPBS, add 50 ul of enzyme-labeled antibody, incubate at RT for 30 min, add 50 ul of chromogenic solution and 50 ul of stop solution. The absorbance value of each sample at a wavelength of 450 nm was measured with Multiskan™ FC System (Thermo Fisher).

### DNA pull-down assay

500 μg of nuclear protein extract, 5 μg of biotin-labeled promoter double-stranded oligonucleotide probe, and 100 μL of streptavidin magnetic beads (Bes5004, Bersinbio, China) were incubated overnight at 4℃. After centrifugation at 5000 × g for 5 min in a precooled fixed-angle centrifuge, the protein-DNA-probe complex was resuspended in 30 μL of loading buffer, and the mixture was boiled for 10 min. The complexes were separated using immunoblotting.

### Flow cytometry analysis or sorting

The cells were resuspended in Flow Cytometry Staining Buffer (00-4222-57, eBioscience), stained with fluorescent dye-conjugated antibodies for 30 min, and washed twice. Samples were collected using the BD FACSAria™ III Cell Sorter and analyzed with FlowJo. During cell sorting, the concentration was maintained at 1 × 10^7^ cells/mL, with a pressure of 60 psi, a 70 μm nozzle, and a maximum rate of 20,000 events/s. Antibodies used in this study: anti-human-CD3E-BV786 (740961, BD Biosciences), anti-human-CD4-FITC (550628, BD Biosciences), anti-human-CD8A-V450 (561426, BD Biosciences), anti-human-CD45-BV510 (563204, BD Biosciences), anti-human-PD1-APC (558694, BD Biosciences), anti-human-TIGIT-PerCP-Cy5.5 (46-9501-82, Invitrogen), anti-human/mouse-TOX (A700-212, Invitrogen), anti-mouse-CD3E-BV786 (417-0031-82, Invitrogen), anti-mouse-CD4-FITC (11-0041-82, Invitrogen), anti-mouse-CD8A-APC (17-0081-82, Invitrogen), anti-mouse-CD36 (MA5-14112, Invitrogen), anti-mouse-CD45-AF700 (56-0451-82, Invitrogen), anti-mouse-CD45-BV510 (567800, BD Biosciences), anti-mouse-CTLA4 (106311, Biolegend, CA, USA), anti-mouse-CTLA4-FITC (HMCD15201, Invitrogen), anti-mouse-PD1-BV421 (404-9981-82, Invitrogen), anti-mouse-PD1-PE (12-9985-82, Invitrogen), anti-mouse-PD1-V450 (75-9981, Cytek, CA, USA), anti-mouse-TIM3-PerCP-Cy5.5 (134011, Biolegend) antibodies. Viability dye used in this study: Fixable Viability Stain 575V (565694, BD Biosciences), and Fixable Viability Stain 780 (565388, BD Biosciences).

### Immunoblotting and immunoprecipitation

The cells were washed with DPBS, lysed using Novex Tricine SDS Sample Buffer (LC1676, Invitrogen) with 1% PMSF (36978, Thermo Scientific), boiled for 20 min, and analyzed by sodium dodecyl sulfate-polyacrylamide gel electrophoresis. For immunoprecipitation, lysate was incubated with primary antibodies for 1 h at 4°C, then with Protein A/G Magnetic Beads (88802, Thermo Scientific) for 2 h at 4°C. The complexes were washed and subjected to electrophoresis. Antibodies used in this study: anti-AMPK (MA5-15815, Invitrogen), anti-CD36 (MA5-14112, Invitrogen), anti-Flag Tag (MA1-91878, Invitrogen), anti-GFP (MA5-15256, Invitrogen), anti-HA Tag (26183, Invitrogen), anti-Histone H3 (PA5-16183, Invitrogen), anti-HMGCR (PA5-37367, Invitrogen), anti-H3S10P (PA5-17869, Invitrogen), anti-INSR (MA5-13783, Invitrogen), anti-IRF4 (14-9858-82, Invitrogen), anti-Myc Tag (PA1-981, Invitrogen), anti-PD1 (14-9969-82, Invitrogen), anti-pAMPK-Ser485 (PA5-117221, Invitrogen), anti-RBPJ (720219, Invitrogen), anti-TNFRSF1B (MA5-32618, Invitrogen), anti-UFL1 (A303-456A, Invitrogen), anti-UFM1 (PA5-90754, Invitrogen), anti-UFSP2 (PA5-99002, Invitrogen), anti-USP7 (PA5-34911, Invitrogen), anti-β-actin (MA1-140, Invitrogen), and anti-6×-His Tag (MA1-21315, Invitrogen) antibodies.

### Immunofluorescence

Cells were fixed with Fixative Solutions (I28800, Thermo Scientifi) and blocked with 5% BSA for 1 h and incubated with anti-H3S10P antibody (PA5-17869, Invitrogen) overnight at 4°C. After three washes with DPBS, cells were incubated with Goat Anti-Rabbit IgG H&L-AF647 antibody (ab150079, Abcam). Photographs were taken with the Vectra3 Automated Quantitative Pathology Imaging system (Akoya Biosciences, MA, USA).

### Isolation of tumor tissue

The primary HCC tissue was diced into 1-3 mm pieces and digested with Tissue Digestion Solution (41423ES10, Yeasen, China) at 37°C for 30 min. EDTA (10 mM, HY-Y0682, MedChemExpress) was added to stop the reaction. The sample was homogenized using a 23 G needle, strained through a 70 mm filter, and centrifuged at 400 × g for 8 min. Then, it was centrifuged at 900 × g for 30 min in 30% Percoll (40501ES60, Yeasen) to isolate the middle white cell layer. The cells were washed twice with DPBS for flow cell sorting.

### Liquid chromatography-mass spectrometry (LC-MS)

Proteins were extracted by 8 M urea (U4883, Merck) with 1% PMSF. Then, the proteins were subjected to Trypsin (HY-129047, MedChemExpress) digestion at an enzyme/protein mass ratio of 1:50 overnight following the Filter-Aided Sample Preparation procedure 26. Specifically, the proteins were subjected to reductive alkylation with Dithiothreitol (D0632, Merck) for 30 min at 56°C and Iodoacetamide (A3221, Merck) for 30 min at RT. Then, the samples were loaded into 10 kDa Centrifugal Filter Unit with Ultracel (MRCPRT010, Millipore), and the urea was diluted and replaced by NH_4_HCO_3_ (5438350100, Merck) gradually after centrifugation for twice with 50 mM NH_4_HCO_3_. Proteins were digested with Trypsin overnight at 37°C. Finally, purified peptide was acquired after extraction with 50% Acetonitrile (34851, Merck) and 0.1% Formic acid (06473, Merck). The peptides were dried in a vacuum at 60°C before LC-MS analysis.

### LC-MS analysis

The peptides were subjected to LC-MS analysis using an Orbitrap Fusion Lumos Tribrid Mass Spectrometer (Thermo Scientific) coupled with an EASY-nLC™ 1000 (Thermo Scientific). Dried peptide was re-suspended in loading buffer and loaded onto a 100 C18 HPLC Columns (100 μm × 2 cm, homemade; particle size, 3 μm; pore size, 120Å, Thermo Scientific) with a maximum pressure of 280 bar using solution A. Then, the peptides were separated on a 100 C18 HPLC Columns (150 μm × 12 cm, homemade; particle size, 1.9 μm; pore size, 120Å) with a gradient of 5 ~ 35% mobile phase B, and adjusted as a series of linear gradients following the different hydrophilic properties of six fractions, respectively, at a flow rate of 600 nl/min for 75 min. The MS analysis was performed by scanning m/z values from 300 to 1400 and a resolution of 120,000 at 200 m/z. An automatic gain control target value of 5 × 10^5^ was used, with a maximum injection time of 50 ms for full scans. The top-speed mode was selected with a 1.6 m/z window and fragmented by higher energy collisional dissociation at a normalized collision energy of 35%. Then measurements were taken using ion trap analyzer with an automatic gain control target of 5 × 10^3^ and a maximum injection time of 35 ms for MS/MS scans. Finally, the dynamic exclusion time was set at 18 s, and data were acquired by Xcalibur 2.2 (Thermo Scientific).

### Peptide and protein identification

MS raw files were processed with the “Firmiana” 27 against the mouse RefSeq protein database. The maximum number of missed cleavages was set to 2. Mass tolerances of 20 ppm for precursor and 0.5 Da for production were allowed. For quality control of protein identification, the target-decoy-based strategy was applied to confirm that the false discovery rate (FDR) of both peptide and protein was lower than 1%. The program percolator was used to obtain probability value (*q* value) and showed that the FDR of every peptide-spectrum match was lower than 1%. Then all peptides shorter than seven amino acids were removed. The cut-off ion score for peptide identification was 20. All the peptide-spectrum matches in all fractions were combined for protein quality control, which was a stringent quality control strategy. The *q* values of both target and decoy peptide sequences were dynamically increased until the corresponding protein FDR was less than 1% employing the parsimony principle. Finally, to reduce the false positive rate, the proteins with at least one unique peptide were selected for further investigation.

### Label-free-based MS quantification of proteins

“Firmiana” 27 was employed for protein quantification. Here, the identification results and the raw data from mzXML file were loaded into the “Firmiana”. Then for each identified peptide, the XIC (extracted-ion chromatogram) was extracted by searching against the MS1 based on its identification information, and the abundance was estimated by calculating the area under the extracted XIC curve. For protein abundance calculation, the nonredundant peptide list was used to assemble proteins following the parsimony principle. Then, the protein abundances were firstly corrected by deploying a traditional label-free, iBAQ algorithm, which used number of theoretical peptides to correct the differences in signal intensity caused by protein size and sequence.

### Mass spectrometry (MS)

Protein bound to USP7 was isolated with anti-USP7 antibody (PA5-34911, Invitrogen) and sent to 10K Genomics (Shanghai, China) for analysis.

### Molecular docking analysis of protein with protein

HDOCK 12 was used as a molecular docking program to analyze the interaction among proteins. PyMol was used to separate the original ligand and protein structure, dehydrate to remove organic matter, and then the Prepare module of Discovery Studio was used to prepare the protein, such as hydrogenation and protonation. LigPlus 28 was performed to analyze the forces between two proteins in two dimensions. The protein interaction interface was analyzed using the Analysis Interface module of Discovery Studio, and PyMol was applied to draw the interacting amino acid residues between two proteins.

### RT-qPCR

Total RNA was extracted using the FastPure Cell/Tissue Total RNA Isolation Kit V2 (RC112-01, Vazyme, China), then reverse transcribed with HiScript II Q Select RT SuperMix (R233-01, Vazyme). RT-qPCR was conducted on Applied Biosystems systems with ChamQ SYBR qPCR Master Mix (Q311-02, Vazyme). The PCR cycle was 95°C for 30s, then 40 cycles of 95°C for 10s, 63°C for 10s, and 72°C for 30s. Gene expression was analyzed using the 2^-ΔΔCT^ method. Primers for mRNA: *Irf4* (F-TCCGACAGTGGTTGATCGAC, R-CCTCACGATTGTAGTCCTGCTT), *Rbpj* (F-ATGCCCTCCGGTTTTCCTC, R-GGACAAGCCCTCCGAGTAGT), *Tnfrsf1b* (F-ACACCCTACAAACCGGAACC, R-AGCCTTCCTGTCATAGTATTCCT), *Usp7* (F-AAGTCTCAAGGTTATAGGGACGG, R-CCATGCTTGTCTGGGTATAGTGT), and β-actin (F-GGCTGTATTCCCCTCCATCG, R-CCAGTTGGTAACAATGCCATGT).

### Transcriptome sequencing

Total RNA was extracted and purified with FastPure Cell/Tissue Total RNA Isolation Kit V2. RNA-seq libraries were constructed with VAHTS® mRNA-seq V3 Library Prep Kit for Illumina (NR611, Vazyme). Sequencing was performed using Illumina NovaSeq 6000 platform (provided by 10K Genomics), and the sequencing depth of each sample was 6G bases.

### Statistical analysis

We used SPSS 19.0 (IBM, NY, USA) and R 4.4 (Lucent Technologies, NJ, USA) software for statistical analysis, and Prism 9.0 (GraphPad, CA, USA) and R 4.4 software to generate visual images. All data were expressed as mean ± SD and reported *P* values less than 0.05 were considered statistically significant. Normality analyses were conducted using Shapiro-Wilk test and D'Agostino and Pearson tests. When comparing two samples, the independent sample *t* test was used if the data were normally distributed, and the variances were homogeneous. Otherwise, Wilcoxon rank sum test was used. Kaplan-Meier curves with 95% confidence intervals were plotted and the Log-rank test was used to compare survival curves.

## Results

### STS alleviated T cell exhaustion in HCC

**Figure 1:**
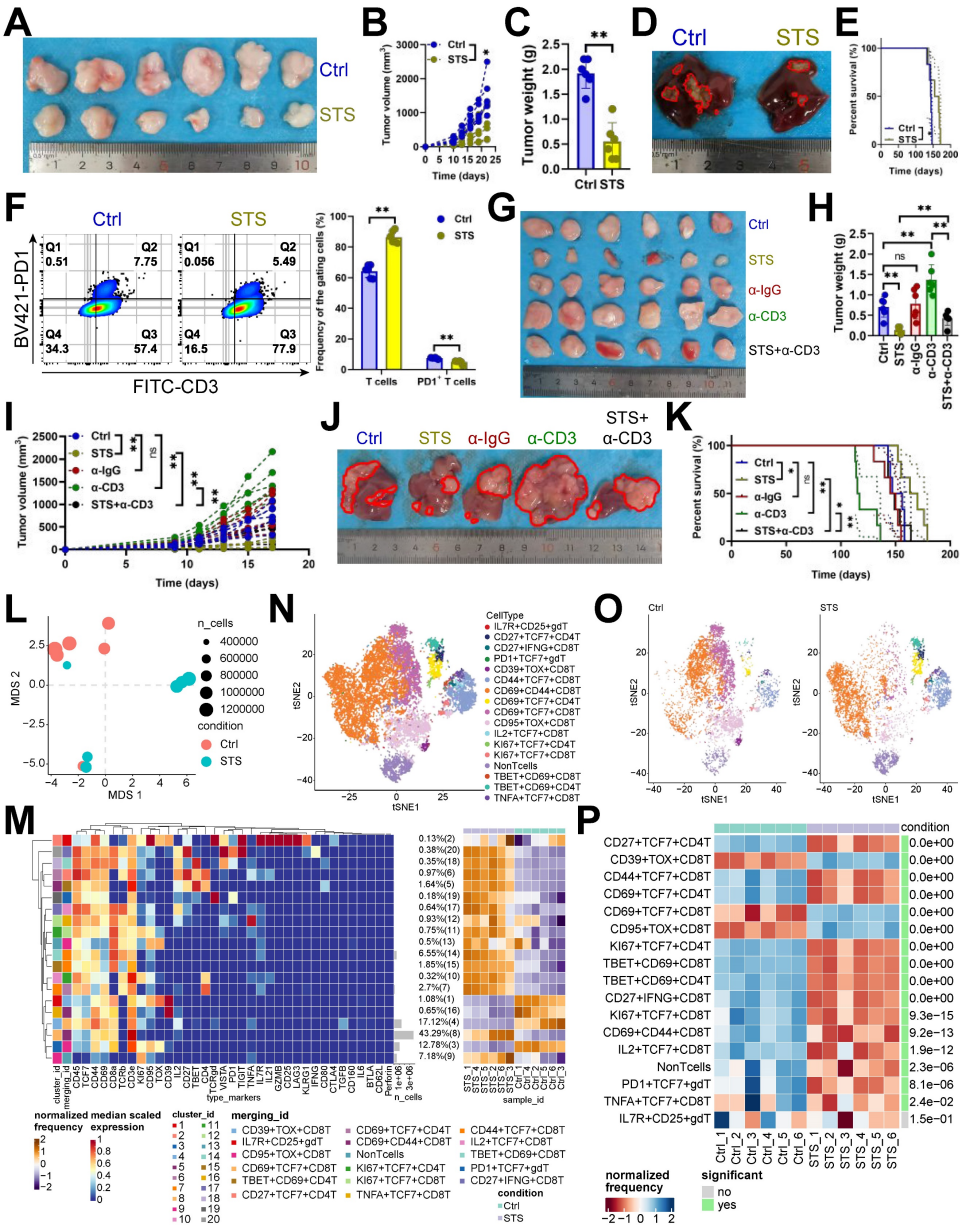
STS alleviated T cell exhaustion.** (A-C)** Effects of STS on subcutaneous tumor growth (*n* = 6). **(A)** Representative. **(B)** Growth curve. **(C)** Tumor weight. **(D, E)** Effects of STS on primary cancer growth (*n* = 6). **(D)** Representative. **(E)** Survival curve. **(F)** Effects of STS on T cell infiltration and its PD1 expression in primary cancer (*n* = 6). **(G-I)** Effects of STS and neutralizing CD3^+^ T cells on subcutaneous tumor growth (*n* = 6). Anti-mouse CD3ε antibody was injected into the tail vein to clear T cells. **(G)** Representative. **(H)** Growth curve. **(I)** Tumor weight. **(J, K)** Effects of STS and neutralizing CD3^+^ T cells on primary cancer growth (*n* = 6). **(J)** Representative. **(K)** Survival curve. **(L)** Non-metric Multidimensional Scaling analysis compared the similarity of T cell characteristic antigen expression before and after STS (*n* = 6). **(M)** Heatmap showed the median expression of the antigen used to generate SOM (*n* = 6). **(N)** SOM was superimposed on mass cytometry data of primary carcinoma-infiltrating T cells (*n* = 6). **(O)** Mosaic of single T cells (*n* = 6). **(P)** Heatmap showed the proportion of T cell subsets (*n* = 6). **(B)**, **(C)**, **(F)**, **(H)**, **(I)**, and **(P)** represented mean ± SD analyzed by unpaired *t* test, **(E)** and **(K)** were analyzed by Log-rank test, **(M)** was analyzed by Euclidean Distance Clustering Algorithm. *P <0.05, **P <0.01. SOM, self-organizing map; STS, short-term starvation.

Recent findings indicated that STS boosts T cell-based immunotherapy 29, 30. We examined STS's effects on T cells infiltrating HCC and found that STS, compared to a standard diet, reduced tumor growth (Figure 1A-1D), improved prognosis (Figure 1E), increased T cell infiltration (Figure 1F, S1A), and decreased PD1 expression in CD3^+^ T cells (Figure 1F). Our study demonstrated that the neutralization of T cells attenuated the tumor-inhibitory effects of STS, suggesting that STS facilitated tumor suppression primarily through the enhancement of T cell function and infiltration (Figure 1G-1K). Furthermore, STS exhibited anticancer properties in tumor-bearing subjects with T cell deficiency (Figure 1G-1K). We hypothesized that this effect may be attributed to the impediment of tumor cell proliferation due to caloric restriction 31. Using mass cytometry, we detailed STS's effects on infiltrating T cells, finding that STS significantly altered T cell function (Figure 1L). Compared with CD4^+^ T cells, CD8^+^ T cells were more significantly affected by STS (Figure S1B). It increased CD3^+^ T cell infiltration and inhibited four exhaustion genes (LAG3, PD1, TOX, and 2B4) expression in CD4^+^ T cells and six exhaustion genes (CD39, CTLA4, PD1, TIGIT, TOX, and VISTA) expression in CD8^+^ T cells (Figure S1C, S1D). It also increased the expression of effector IL21 in CD4^+^ or CD8^+^ T cells and proliferation marker KI67 in CD4^+^ T cells (Figure S1C, S1D).

Hierarchical clustering heatmap identified 16 T cell subsets (Figure 1M), including IL7R^+^ CD25^+^ γδT cells, CD27^+^ TCF7^+^ CD4^+^ T cells, CD27^+^ IFNG^+^ CD8^+^ T cells, PD1^+^ TCF7^+^ γδT cells, CD39^+^ TOX^+^ CD8^+^ T cells, CD44^+^ TCF7^+^ CD8^+^ T cells, CD69^+^ CD44^+^ CD8^+^ T cells, CD69^+^ TCF7^+^ CD4^+^ T cells, CD69^+^ TCF7^+^ CD8^+^ T cells, CD95^+^ TOX^+^ CD8^+^ T cells, IL2^+^ TCF7^+^ CD8^+^ T cells, KI67^+^ TCF7^+^ CD4^+^ T cells, KI67^+^ TCF7^+^ CD8^+^ T cells, TBET^+^ CD69^+^ CD8^+^ T cells, TBET^+^ CD69^+^ CD4^+^ T cells, and TNFA^+^ TCF7^+^ CD8^+^ T cells (Figure 1N). Post-STS, CD27^+^ TCF7^+^ CD4^+^ T cells, CD44^+^ TCF7^+^ CD8^+^ T cells, CD69^+^ TCF7^+^ CD4^+^ T cells, KI67^+^ TCF7^+^ CD4^+^ T cells, TBET^+^ CD69^+^ CD8^+^ T cells, TBET^+^ CD69^+^ CD4^+^ T cells, CD27^+^ IFNG^+^ CD8^+^ T cells, KI67^+^ TCF7^+^ CD8^+^ T cells, CD69^+^ CD44^+^ CD8^+^ T cells, IL2^+^ TCF7^+^ CD8^+^ T cells, PD1^+^ TCF7^+^ γδT cells, TNFA^+^ TCF7^+^ CD8^+^ T cells increased, and CD39^+^ TOX^+^ CD8^+^ T cells, CD69^+^ TCF7^+^ CD8^+^ T cells, CD95^+^ TOX^+^ CD8^+^ T cells decreased (Figure 1O, 1P).

Additionally, we investigated the impact of STS-stimulated and STS-pre-stimulated tumor cells on T cell exhaustion *in vitro* to analyze whether STS directly regulates T cells or indirectly influences T cells through tumor cells. Our findings indicated that both conditions inhibited the expression of inhibitory receptors on T cells (Figure S2A-S2C). We first investigated why STS-pre-stimulated tumor cells suppressed inhibitory receptor expression on T cells. Given that AMPK activation leads to the phosphorylation of PDL1 at Ser283, resulting in its degradation 32, and that an insufficient energy supply inevitably activates the AMPK pathway 33, we proceeded to knock out *Ampk* and *Pdl1* in Hep-53.4 cells. Our results demonstrated that *Ampk*-KO tumor cells up-regulated the expression of PD1 and TIM3 on CD8^+^ T cells, which were inhibited by STS, while *Pdl1*-KO tumor cells reduced the elevated expression of PD1 and TIM3 on CD8^+^ T cells induced by *Ampk*-KO (Figure S2C-S2D). These findings suggested that STS inhibited PDL1 expression by activating AMPK phosphorylation in tumor cells, thereby protecting T cells from exhaustion. Next, we investigated the molecular mechanisms by which STS protected T cells from dysfunction from the perspective of T cells themselves.

### STS alleviated T cell exhaustion by inhibiting USP7

**Figure 2:**
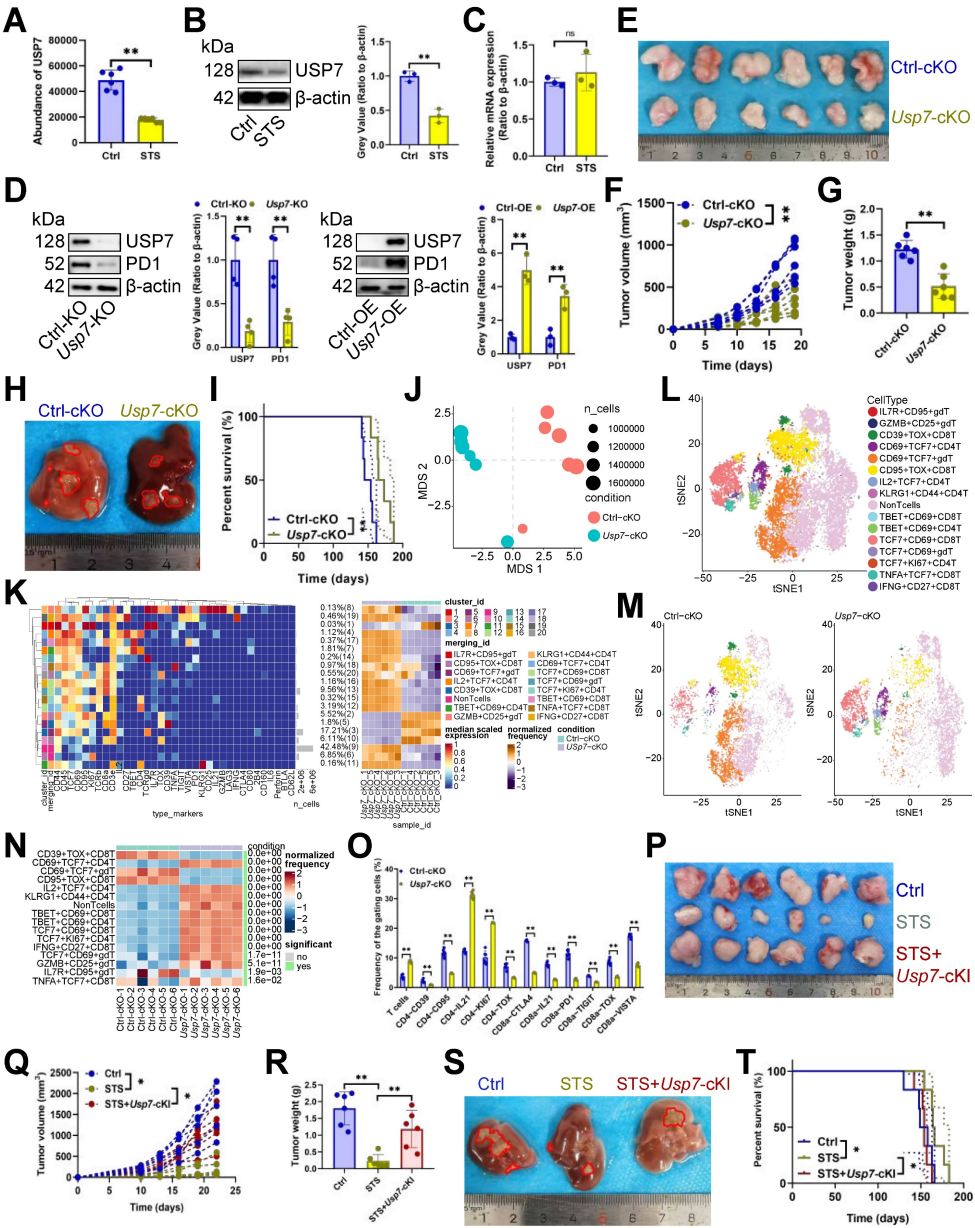
STS alleviated T cell exhaustion by inhibiting USP7.** (A-C)** Effects of STS on USP7 expression in primary carcinoma-infiltrating CD3^+^ T cells were analyzed by mass spectrometry **(A)**, immunoblotting **(B)** and RT-qPCR **(C)** (*n* = 6). **(D)** Effects of KO or OE of *Usp7* on PD1 expression in CD3^+^ T cells (*n* = 6). (E-G) Effects of *Usp7*-cKO on subcutaneous tumor growth (*n* = 6). **(E)** Representative. **(F)** Growth curve. **(G)** Tumor weight. (H, I) Effects of *Usp7*-cKO on primary cancer growth (*n* = 6). **(H)** Representative. **(I)** Survival curve. **(J)** Non-metric Multidimensional Scaling analysis compared the similarity of T cell characteristic antigen expression before and after *Usp7*-cKO (*n* = 6). **(K)** Heatmap showed the median expression of the antigen used to generate SOM (*n* = 6). **(L)** SOM was superimposed on mass cytometry data of primary carcinoma-infiltrating T cells (*n* = 6). **(M)** Mosaic of single T cells (*n* = 6). **(N)** Heatmap showed the proportion of T cell subsets (*n* = 6). **(O)** Median expression of T cell characteristic antigen (*n* = 6). **(P-R)** Effects of STS and *Usp7*-cKI on subcutaneous tumor growth (n = 6). **(P)** Representative. **(Q)** Growth curve. **(R)** Tumor weight. **(S, T)** Effects of STS and *Usp7*-cKI on primary carcinoma growth (*n* = 6). **(S)** Representative. **(T)** Survival curve. (A-D), **(F)**, **(G)**, **(N)**, **(O)**, **(Q)**, and **(R)** represented mean ± SD analyzed by unpaired *t* test, **(I)** and **(T)** were analyzed by Log-rank test, **(K)** was analyzed by Euclidean Distance Clustering Algorithm. *P <0.05, **P <0.01. cKI, conditional knock-in; cKO, conditional knockout; OE, overexpression; SOM, self-organizing map; STS, short-term starvation.

The oncogenic potential of TME is modulated by the interplay between nutritional deficiencies and ubiquitin proteasome systems 34. Nonetheless, the interaction between STS and deubiquitinase in T cells remains unexplored. We investigated STS's effects on deubiquitinase expression. Mass spectrometry (MS) and immunoblotting assays revealed that STS exclusively inhibited USP7 expression in CD3^+^ T cells (Figure 2A, 2B) without affecting its transcription (Figure 2C). It is essential to first ascertain whether STS suppressed USP7 expression in tumor cells and to understand the subsequent impact on the susceptibility of these cells to T cell-mediated cytotoxicity. Previous research has demonstrated that USP7 plays a protective role in preventing the degradation of PDL1, thereby reducing the susceptibility of tumor cells to T cell-mediated cytotoxicity 35. Consequently, it is imperative to ascertain whether STS inhibit USP7 expression in tumor cells to potentiate T cell function. Our findings indicated that STS suppressed USP7 expression in Hep-53.4 cells (Figure S3A), and the KO of *Usp7* in Hep-53.4 cells resulted in inhibited tumor growth (Figure S3B-S3D), with the inverse also being true (Figure S3E-S3G). Moreover, *Usp7*-KO in Hep-53.4 cells led to reduced PDL1 expression (Figure S3H), and the reverse was observed as well (Figure S3I). Rescue experiments revealed that STS diminished the expression of both USP7 and PDL1 in Hep-53.4 cells, while *Usp7*-OE mitigated the inhibitory effect of STS on PDL1 expression (Figure S3J). *In vivo* studies further demonstrated that STS impeded tumor growth, and this inhibitory effect was attenuated by *Usp7*-OE in tumor cells (Figure S3K-S3M). Additionally, the tumor-promoting effect of *Usp7*-OE was counteracted by *Pdl1*-KO (Figure S3N-S3P), suggesting that STS exerted its effects by inhibiting USP7, thereby reducing PDL1 expression and enhancing the sensitivity of tumor cells to T cell-mediated killing. Following this, we investigated the role of USP7 in T cells, specifically focusing on its influence on T cell exhaustion.

Our study indicated that overexpressing *Usp7* increased PD1 expression in CD3^+^ T cells, whereas inhibiting it reduced PD1 levels (Figure 2D). *In vivo*, *Usp7*-cKO suppressed tumor growth (Figure 2E-2H) and improved prognosis (Figure 2I). Mass cytometry was also used to detail *Usp7*-cKO's regulatory impact on T cells, and non-metric Multidimensional Scaling (NMDS) analysis indicated that *Usp7*-cKO significantly changed T cell function (Figure 2J). Hierarchical clustering heatmap identified 15 T cell subsets (Figure 2K), including IL7R^+^ CD95^+^ γδT cells, GZMB^+^ CD25^+^ γδT cells, CD39^+^ TOX^+^ CD8^+^ T cells, CD69^+^ TCF7^+^ CD4^+^ T cells, CD69^+^ TCF7^+^ γδT cells, CD95^+^ TOX^+^ CD8^+^ T cells, IL2^+^ TCF7^+^ CD4^+^ T cells, KLRG1^+^ CD44^+^ CD4^+^ T cells, TBET^+^ CD69^+^ CD8^+^ T cells, TBET^+^ CD69^+^ CD4^+^ T cells, TCF7^+^ CD69^+^ CD8^+^ T cells, TCF7^+^ CD69^+^ γδT cells, TCF7^+^ KI67^+^ CD4^+^ T cells, TNFA^+^ TCF7^+^ CD8^+^ T cells, and IFNG^+^ CD27^+^ CD8^+^ T cells (Figure 2L). Post-*Usp7* loss, CD69^+^ TCF7^+^ CD4^+^ T cells, IL2^+^ TCF7^+^ CD4^+^ T cells, KLRG1^+^ CD44^+^ CD4^+^ T cells, TBET^+^ CD69^+^ CD8^+^ T cells, TBET^+^ CD69^+^ CD4^+^ T cells, TCF7^+^ CD69^+^ CD8^+^ T cells, TCF7^+^ KI67^+^ CD4^+^ T cells, IFNG^+^ CD27^+^ CD8^+^ T cells, TCF7^+^ CD69^+^ γδT cells, GZMB^+^ CD25^+^ γδT cells, TNFA^+^ TCF7^+^ CD8^+^ T cells increased, while CD39^+^ TOX^+^ CD8^+^ T cells, CD69^+^ TCF7^+^ γδT cells, CD95^+^ TOX^+^ CD8^+^ T cells, IL7R^+^ CD95^+^ γδT cells decreased (Figure 2M, 2N). In addition, it increased CD3^+^ T cell infiltration and inhibited three exhaustion genes (CD39, CD95, and TOX) expression in CD4^+^ T cells and five exhaustion genes (CTLA4, PD1, TIGIT, TOX, and VISTA) expression in CD8^+^ T cells (Figure 2O, S3Q). It also increased the expression of IL21 in CD4^+^ or CD8^+^ T cells and KI67 in CD4^+^ T cells (Figure 2O, S3Q). Furthermore, we used mice *Usp7*-cKI to analyze if STS's anticancer effects rely on USP7 inhibition. As expected, *Usp7*-cKI reduced STS's anti-tumor efficacy (Figure 2P-2T).

### USP7 inhibited RBPJ ubiquitination

**Figure 3:**
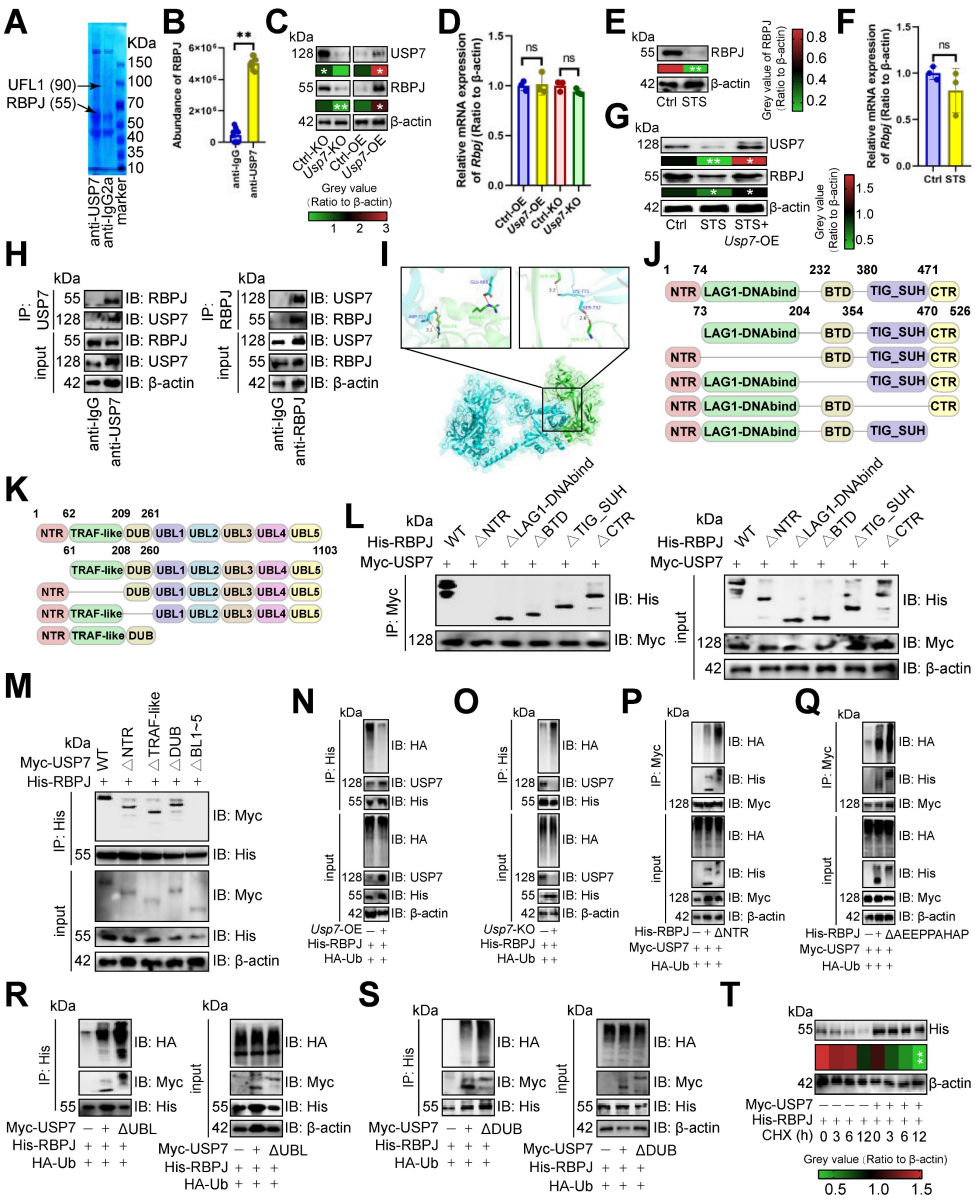
USP7 inhibited RBPJ ubiquitination. **(A)** Identification of proteins bound by USP7 in primary carcinoma-infiltrating CD3^+^ T cells by immunoprecipitation-mass spectrometry assays. **(B)** PXD039633 dataset showed the binding of USP7 and RBPJ (*n* =10). **(C, D)** Effects of OE or KO of *Usp7* on the expression of RBPJ protein **(C)** and transcript **(D)** in CD3^+^ T cells (*n* = 3). **(E, F)** Effects of STS on the expression of RBPJ protein **(E)** and transcript **(F)** in CD3^+^ T cells (*n* = 3). **(G)** Effects of STS and *Usp7*-OE on the RBPJ protein levels in CD3^+^ T cells (*n* = 3). **(H)** CD3^+^ T cell lysate was treated with anti-IgG control and -USP7 (left) or -RBPJ (right) antibodies, with 5% lysate as input control (*n* = 3). **(I)** Surface plot presented docking models and interface residues between USP7 (sky blue) and RBPJ (green) proteins, hydrogen bonds highlighted with dashed lines. **(J, K)** Schematic plot of domain-deficient mutants for USP7 **(J)** and RBPJ **(K)**. **(L, M)** Immunoprecipitation identified key domains of USP7 and RBPJ binding (*n* = 3). CD3^+^ T cells were co-transfected with WT or domain-deficient mutants **(L)** of Myc-labeled USP7 and His-labeled RBPJ, and vice versa **(M)**. **(N, O)** Effects of OE **(N)** or KO **(O)** of *Usp7* on RBPJ ubiquitination in CD3^+^ T cells (*n* = 3). **(P, Q)** Effects of the NTR **(P)** and AEEPPAHAP **(Q)** of RBPJ on its ubiquitination in CD3^+^ T cells (*n* = 3). **(R, S)** Effect of the UBL domain **(R)** and the DUB domain **(S)** of USP7 on RBPJ ubiquitination in CD3^+^ T cells (*n* = 3). **(T)** CD3^+^ T cells with His-labeled RBPJ were treated with Cycloheximide, and their expression was evaluated (*n* = 3). **(B-G)**, and **(T)** represented mean ± SD analyzed by unpaired *t* test. *P <0.05, **P <0.01. KO, knockout; OE, overexpression; STS, short-term starvation; WT, wild-type.

Since USP7 is a deubiquitinase 36, we conducted immunoprecipitation-MS assays to identify its substrate affecting T cell function. Our analysis revealed an absence of binding between USP7 and PD1 (Figure 3A). Consequently, we sought to identify the protein whose ubiquitination degradation is inhibited by USP7 to sustain PD1 expression. We found that USP7 bound to RBPJ in CD3^+^ T cells, a T cell exhaustion protein 37(Figure 3A), which was confirmed by earlier data 38 (Figure 3B). Our study demonstrated that *Usp7*-KO led to the inhibition of RBPJ protein expression in CD3^+^ T cells, and *Usp7*-OE up-regulated it (Figure 3C), with no impact on its transcript levels (Figure 3D). This suggested that USP7 played a role in inhibiting the RBPJ ubiquitination in CD3^+^ T cells. Additionally, STS, which acted upstream of USP7, was found to inhibit RBPJ expression at the translational level (Figure 3E) rather than the transcriptional level (Figure 3F) in CD3^+^ T cells. Rescue experiments further revealed that STS diminished the expression of both USP7 and RBPJ in T cells, while *Usp7*-OE mitigated the inhibitory effect of STS on RBPJ expression (Figure 3G). Subsequently, we investigated the interaction between USP7 and RBPJ. Through immunoprecipitation and molecular docking analyses, we confirmed the interaction between them in CD3^+^ T cells (Figure 3H, 3I). To further elucidate the binding domains, we synthesized a series of domain-deficient mutants of them (Figure 3J, 3K) and discovered that the N-terminal region (NTR) of RBPJ interacted with the UBL1-5 domain of USP7 in CD3^+^ T cells (Figure 3L, 3M).

We then investigated USP7's role in RBPJ protein degradation. *Usp7*-OE appeared to reduce RBPJ ubiquitination in CD3^+^ T cells (Figure 3N), while its absence increased it (Figure 3O). The NTR of RBPJ was crucial for complex formation, and its deletion boosted RBPJ ubiquitination in CD3^+^ T cells (Figure 3P). The AXXPXAXAP motif is key for DUB recognition in RBPJ 39, and removing it was found to enhance RBPJ ubiquitination in CD3^+^ T cells (Figure 3Q). The UBL1-5 domain of USP7 was also essential for binding, and its absence increased RBPJ ubiquitination in CD3^+^ T cells (Figure 3R). It has been reported that the DUB domain binds ubiquitin 36, and its absence was shown to increase RBPJ ubiquitination in CD3^+^ T cells (Figure 3S). Finally, we indicated that overexpressing *Usp7*, while using Cycloheximide to accelerate protein degradation, slowed RBPJ degradation in CD3^+^ T cells (Figure 3T).

### USP7 aggravated T cell exhaustion by up-regulating RBPJ

**Figure 4:**
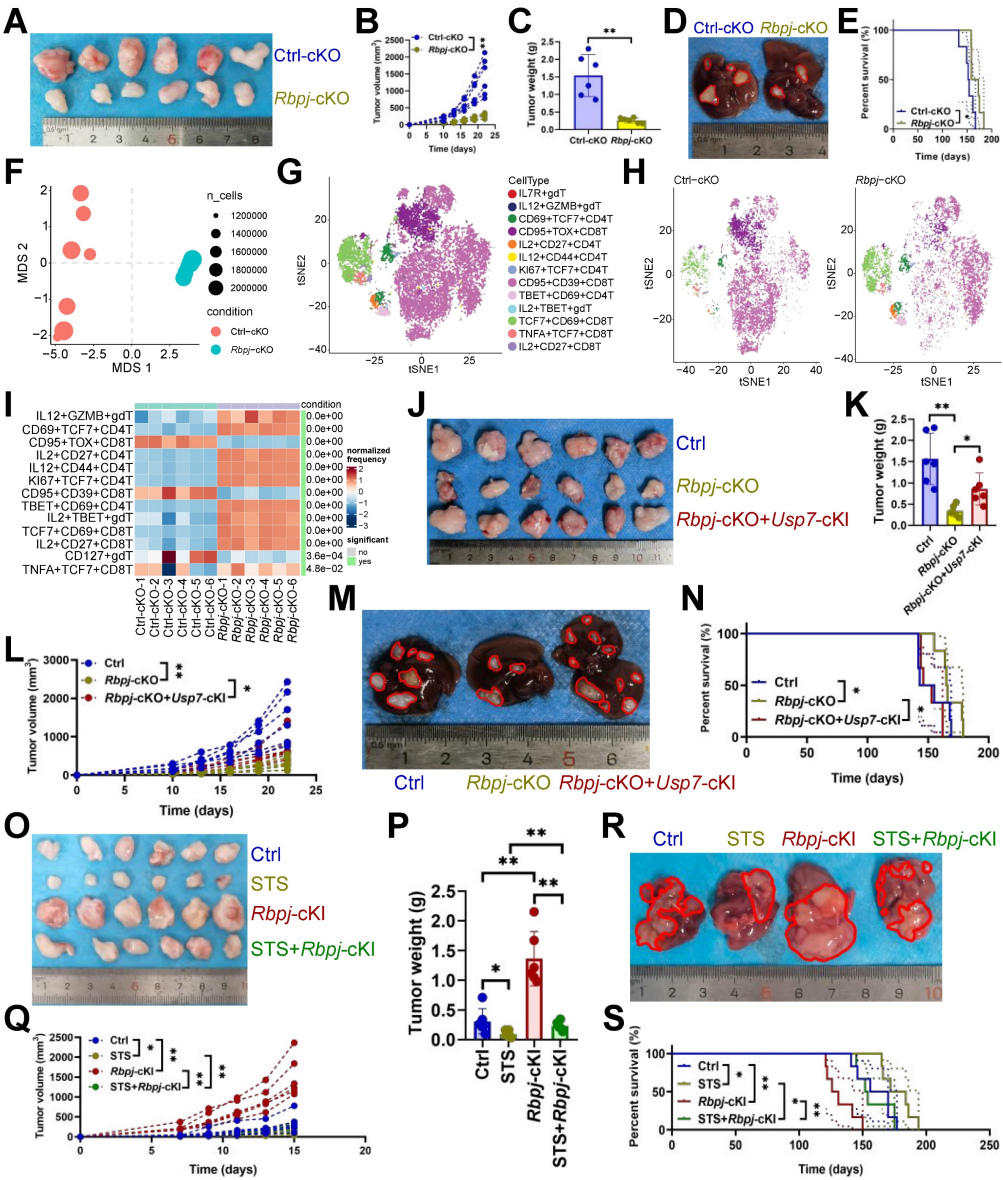
USP7 aggravated T cell exhaustion by up-regulating RBPJ. **(A-C)** Effects of *Rbpj*-cKO on subcutaneous tumor growth (*n* = 6). **(A)** Representative. **(B)** Growth curve. **(C)** Tumor weight. **(D, E)** Effects of *Rbpj*-cKO on primary cancer growth (*n* = 6). **(D)** Representative. **(E)** Survival curve. **(F)** Non-metric Multidimensional Scaling analysis compared the similarity of T cell characteristic antigen expression before and after *Rbpj*-cKO (*n* = 6). **(G)** SOM was superimposed on mass cytometry data of primary carcinoma-infiltrating T cells (*n* = 6). **(H)** Mosaic map of single T cells (*n* = 6). **(I)** Heatmap showed the proportion of T cell subsets (n = 6). **(J-L)** Effects of *Rbpj*-cKO and *Usp7*-cKI on subcutaneous tumor growth (*n* = 6). **(J)** Representative. **(K)** Growth curve. **(L)** Tumor weight. **(M, N)** Effects of *Rbpj*-cKO and *Usp7*-cKI on primary cancer growth (*n* = 6). **(M)** Representative. **(N)** Survival curve. **(O-Q)** Effects of STS and *Rbpj*-cKI on subcutaneous tumor growth (*n* = 6). **(O)** Representative. **(P)** Growth curve. **(Q)** Tumor weight. **(R, S)** Effects of STS and *Rbpj*-cKI on primary cancer growth (*n* = 6). **(R)** Representative. **(S)** Survival curve. **(B)**, **(C)**, **(I)**, **(K)**, **(L)**, **(P)**, and **(Q)** represented mean ± SD analyzed by unpaired *t* test, **(E)**, **(N)**, and **(S)** were analyzed by Log-rank test. *P <0.05, **P <0.01. cKI, conditional knock-in; cKO, conditional knockout; SOM, self-organizing map.

We analyzed the effect of *Rbpj*-cKO on HCC and revealed it inhibited tumor growth (Figure 4A-4D) and improved prognosis (Figure 4E). Mass cytometry was performed to detail *Rbpj*-cKO's regulatory effect on T cells. NMDS analysis indicated that *Rbpj*-cKO significantly changed T cell function (Figure 4F). Hierarchical clustering heatmap identified 13 T cell subsets (Figure S4A), including IL7R^+^ γδT cells, IL12^+^ GZMB^+^ γδT cells, CD69^+^ TCF7^+^ CD4^+^ T cells, CD95^+^ TOX^+^ CD8^+^ T cells, IL2^+^ CD27^+^ CD4^+^ T cells, IL12^+^ CD44^+^ CD4^+^ T cells, KI67^+^ TCF7^+^ CD4^+^ T cells, CD95^+^ CD39^+^ CD8^+^ T cells, TBET^+^ CD69^+^ CD4^+^ T cells, IL2^+^ TBET^+^ γδT cells, TCF7^+^ CD69^+^ CD8^+^ T cells, TNFA^+^ TCF7^+^ CD8^+^ T cells, and IL2^+^ CD27^+^ CD8^+^ T cells (Figure 4G). Post-*Rbpj*-cKO, IL12^+^ GZMB^+^ γδT cells, CD69^+^ TCF7^+^ CD4^+^ T cells, IL2^+^ CD27^+^ CD4^+^ T cells, IL12^+^ CD44^+^ CD4^+^ T cells, KI67^+^ TCF7^+^ CD4^+^ T cells, TBET^+^ CD69^+^ CD4^+^ T cells, IL2^+^ TBET^+^ γδT cells, TCF7^+^ CD69^+^ CD8^+^ T cells, IL2^+^ CD27^+^ CD8^+^ T cells, and TNFA^+^ TCF7^+^ CD8^+^ T cells increased, while CD95^+^ TOX^+^ CD8^+^ T cells, CD95^+^ CD39^+^ CD8^+^ T cells, and IL7R^+^ γδT cells decreased (Figure 4H, 4I). Moreover, *Rbpj*-cKO appeared to increase CD3^+^ T cell infiltration and inhibited two exhaustion genes (CTLA4 and TOX) expression in CD4^+^ T cells and five exhaustion genes (CD39, CTLA4, PD1, TOX, and VISTA) expression in CD8^+^ T cells (Figure S4B). It also increased the expression of IL21 in CD4^+^ or CD8^+^ T cells and KI67 in CD4^+^ T cells (Figure S4B). What's more, *Usp7*-cKI was witnessed to reduce the antitumor efficacy of *Rbpj*-cKO (Figure 4J-4N), and *Rbpj*-cKI rescued the tumor reduction phenotype under STS treatment (Figure 4O-4S).

### RBPJ enhanced transcription of exhaustion genes

**Figure 5:**
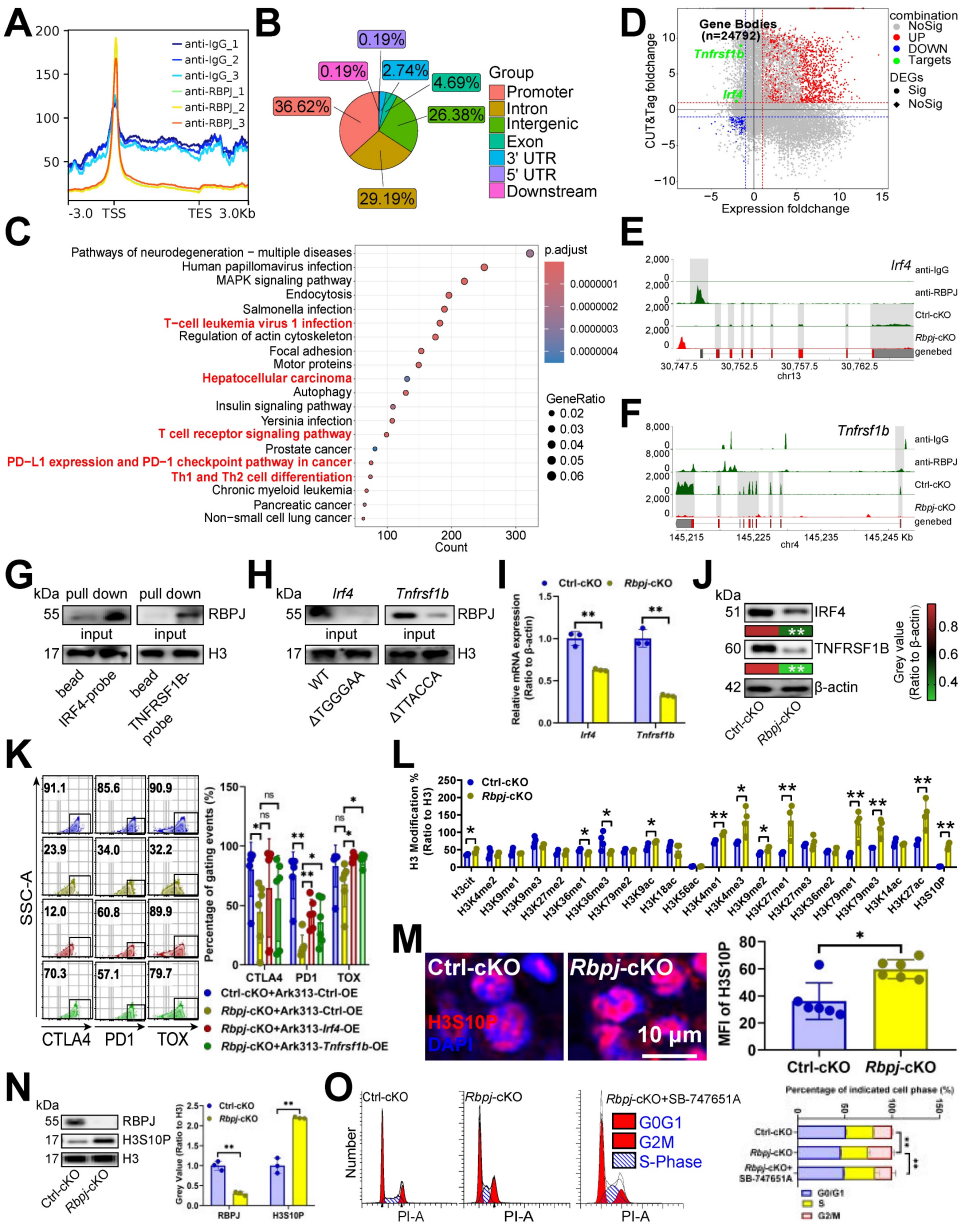
RBPJ enhanced transcription of exhaustion genes. **(A, B)** The cluster plot showed the DNA fragments that RBPJ binds to **(A)**, and their distribution across the genome **(B)** in primary carcinoma-infiltrating CD3^+^ T cells (*n* = 3). **(C)** Pathway enrichment analysis of genes mapped to RBPJ-bound DNA fragments compared to IgG controls in CD3^+^ T cells (*n* = 3). Red indicated T cell- or HCC-related items. **(D)** A four-quadrant plot presented the distribution of genes with significant alterations in both the DNA fragment bound to RBPJ and the mRNA levels in CD3^+^ T cells (*n* = 3). (E, F) Snapshot plots showed explicit transcription expression of *Irf4* **(E)** and *Tnfrsf1b* **(F)** and the enrichment signal of RBPJ on their promoter in CD3^+^ T cells (*n* = 3). Gray indicated the differential signal. **(G)** DNA pull-down experiment demonstrated the binding of RBPJ to the promoter of *Irf4* or *Tnfrsf1b* in CD3^+^ T cells (*n* = 3). **(H)** Effects of loss of TGGGAA of *Irf4* promoter or TTACCA of *Tnfrsf1b* promoter on DNA affinity of RBPJ in CD3^+^ T cells (*n* = 3). **(I, J)** Effects of *Rbpj*-cKO on transcription **(I)** and protein **(J)** expression of IRF4 or TNFRSF1B in CD3^+^ T cells (*n* = 3). **(K)** Flow cytometry demonstrated inhibitory receptor expression in CD3^+^ T cells (*n* = 6). **(L)** ELISA demonstrated 22 histone H3 modification levels in CD3^+^ T cells (*n* = 4). (M, N) Immunofluorescence **(M)** and immunoblotting **(N)** demonstrated the expression of H3S10P in CD3^+^ T cells (*n* = 3). **(O)** Flow cytometry presented the cell cycle of CD3^+^ T cells (*n* = 3). **(I-O)** represented mean ± SD analyzed by unpaired *t* test. *P <0.05, **P <0.01. cKO, conditional knockout.

Given that Notch1 causes T cell failure 40, we evaluated RBPJ's dependence on Notch1 for T cell regulation. Notch signaling inhibitors MK-0752 and Avagacestat alleviated T cell exhaustion, and *Rbpj*-cKO further reduced exhaustion gene expression in T cells (Figure S5A), suggesting that RBPJ-induced exhaustion was not entirely dependent on Notch1. To explore RBPJ's role in T cell exhaustion, we analyzed its DNA binding and discovered that RBPJ was bound to transcription start sites and promoters in CD3^+^ T cells (Figure 5A, 5B), including Notch pathway target gene *Ccnd3*, etc. (Figure S5B). KEGG analysis indicated that T cell- or HCC-related items like “T-cell leukemia virus 1 infection”, “Hepatocellular carcinoma”, “T cell receptor signaling pathway”, “PD-L1 expression and PD-1 checkpoint pathway in cancer”, and “Th1 and Th2 cell differentiation” were up-regulated at RBPJ enrichment peaks (Figure 5C). To identify the target of RBPJ induced exhaustion, we integrated RBPJ enrichment peaks, transcripts downregulated by* Rbpj*-cKO , and known T cell exhaustion genes 41, revealing *Irf4* 10 and *Tnfrsf1b* 11 as targets (Figure 5D-5F). DNA pull-down assay confirmed RBPJ's presence on their promoters in CD3^+^ T cells (Figure 5G). JASPAR 42 predicted RBPJ bound to TGGGAA of the *Irf4* promoter and TTACCA of the *Tnfrsf1b* promoter, with mutations at these sites inhibiting their binding in CD3^+^ T cells (Figure 5H). *Rbpj*-cKO appeared to reduce expression of both genes in CD3^+^ T cells (Figure 5I, 5J). Furthermore, overexpression of *Irf4* and *Tnfrsf1b* in CD3^+^ T cells reduced the protective effect of *Rbpj*-cKO, indicating RBPJ induced T cell exhaustion through them (Figure 5K, S5C).

We noted that KI67 expression increased with STS, *Usp7*-cKO, or *Rbpj*-cKO, but RBPJ didn't affect proliferation-related gene transcription (Figure 5D). Given RBPJ's involvement in chromatin conformation and genome transcription via histone H3 modification 43, we analyzed 22 histone H3 modifications, and found H3cit, H3K36me1, H3K36me3, H3K9ac, H3K4me1, H3K4me3, H3K9me2, H3K27me1, H3K79me1, H3K79me3, H3K27ac, and H3S10P levels were elevated after *Rbpj*-cKO, with H3Ser10P (associated with mitotic chromosome condensation 44) being the most significant. Moreover, *Rbpj*-cKO was found to increased H3Ser10P expression in CD3^+^ T cells (Figure 5M, 5N), and the G2/M phase rise caused by *Rbpj*-cKO was reversed by SB-747651A, a H3S10P phosphorylase MSK1 45 inhibitor (Figure 5O). Thus, RBPJ functioned as a transcription factor and chromatin regulator, worsening T cell function and hindering proliferation.

### USP7 underwent UFMylation

**Figure 6:**
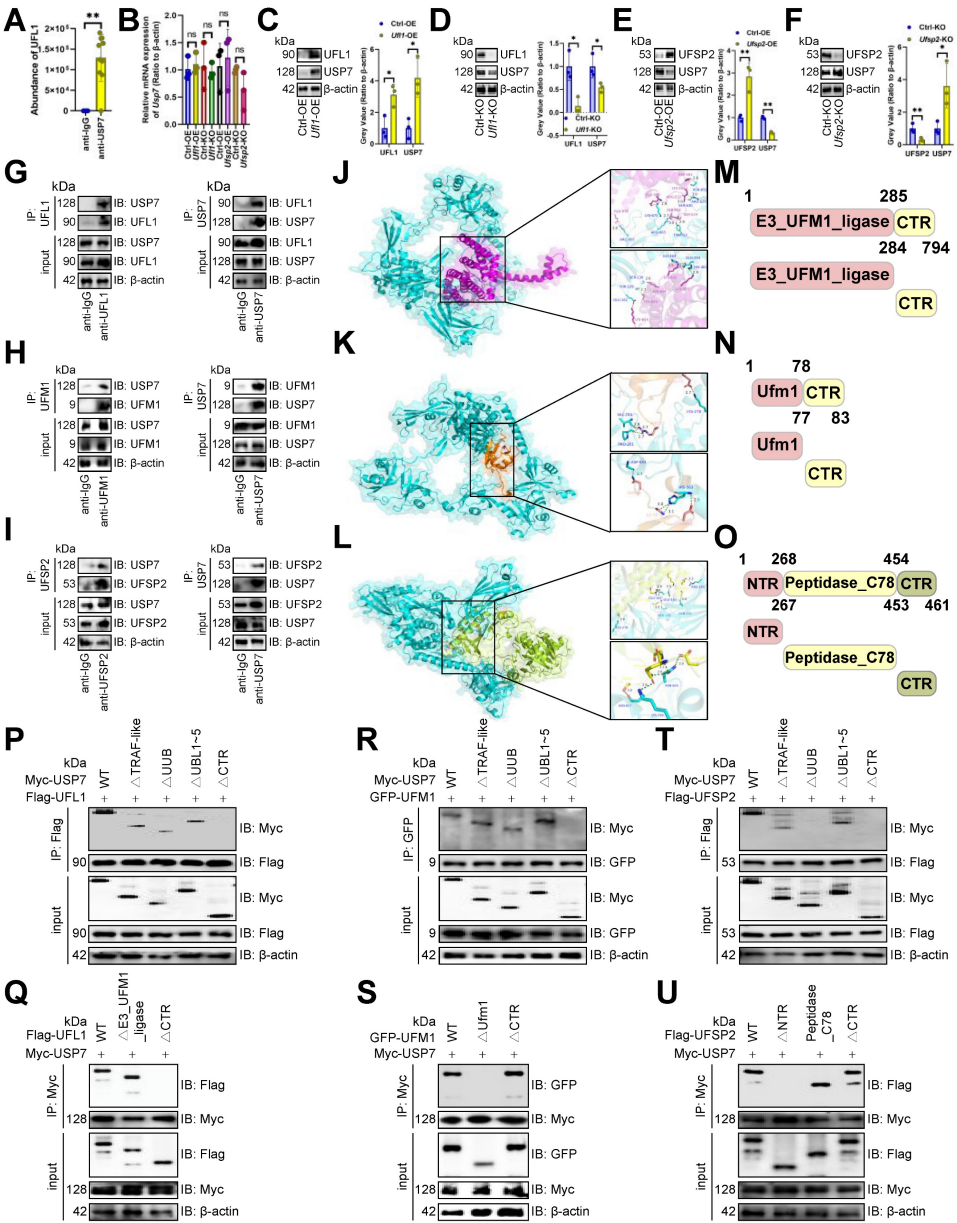
USP7 underwent UFMylation.** (A)** PXD039633 dataset showed the binding of USP7 and UFL1 (*n* =10). **(B)** Effects of OE or KO of *Ufl1* or *Ufsp2* on *Usp7* transcript expression in CD3^+^ T cells (*n* = 3). **(C-F)** Effects of OE **(C, E)** or KO **(D, F)** of *Ufl1* **(C, D)** or *Ufsp2* **(E, F)** on USP7 protein expression in CD3^+^ T cells (*n* = 3). **(G)** CD3^+^ T cell lysate was treated with IgG control and UFL1 (left) or USP7 (right) antibodies, and 5% lysate was used as input control (*n* =3). **(H)** CD3^+^ T cell lysate was treated with anti-IgG controls and -UFM1 (left) or -USP7 (right) antibodies (*n* =3). **(I)** CD3^+^ T cell lysate was treated with anti-IgG controls and -UFSP2 (left) or -USP7 (right) antibodies (*n* =3). **(J-L)** Surface plot presented docking models and interface residues between USP7 (sky blue) and UFL1 (purple) **(J)**/UFM1 (orange) **(K)**/UFSP2 (green) **(L)** proteins, with hydrogen bonds highlighted by dashed lines. **(M-O)** Schematic plot of the domain-deficient mutants of UFL1 **(M)**, UFM1 **(N)**, and UFSP2 **(O)**. **(P, Q)** Immunoprecipitation identified key domains of USP7 and UFL1 binding (*n* =3). CD3^+^ T cells were co-transfected with WT or domain-deficient mutants of Flag-labeled UFL1 and Myc-labeled USP7 **(P)** and vice versa **(Q)**. **(R, S)** Immunoprecipitation identified key domains of USP7 and UFM1 binding (*n* =3). CD3^+^ T cells were co-transfected with WT or domain-deficient mutants of GFP-labeled UFM1 and Myc-labeled USP7 **(R)** and vice versa **(S)**. **(T, U)** Immunoprecipitation identified key domains of USP7 and UFSP2 binding (*n* =3). CD3^+^ T cells were co-transfected with Flag-labeled UFSP2 and Myc-labeled WT or domain-deficient mutants of USP7 **(T)** and vice versa **(U)**. **(A-F)** represented mean ± SD analyzed by unpaired *t* test. *P <0.05, **P <0.01. KO, knockout; OE, overexpression; WT, wild-type.

In view of STS inhibiting the expression of USP7 from translation, we employed immunoprecipitation-MS assays to identify proteins interacting with USP7. USP7 was presented to interact with the UFMylation E3 ligase UFL1 in CD3^+^ T cells 5 (Figure 3A, 6A), suggesting USP7's involvement in UFMylation. We found that although altering *Ufl1* or* Ufsp2* 5 didn't affect USP7 transcripts (Figure 6B), *Ufl1* increased USP7 protein levels (Figure 6C, 6D), and *Ufsp2* decreased them in CD3^+^ T cells (Figure 6E, 6F). Therefore, we believed that USP7 undergoes UFMylation. To explore USP7 UFMylation's molecular mechanism, we examined the binding of UFM1 5, UFL1, and UFSP2 each to USP7. Immunoprecipitation and molecular docking revealed that USP7 bound to UFL1, UFM1, and UFSP2 in CD3^+^ T cells (Figure 6G-6L). To pinpoint the binding regions, we created domain-deficient mutants of them (Figure 6M-6O) and discovered that USP7's C-terminal region (CTR) was bound to UFL1's CTR (Figure 6P, 6Q) and UFM1's Ufm1 domain in CD3^+^ T cells (Figure 6R, 6S). Additionally, USP7's UUB domain and CTR interacted with UFSP2's NTR in CD3^+^ T cells (Figure 6T, 6U).

### STS inhibited USP7 UFMylation thus increasing its ubiquitination

**Figure 7:**
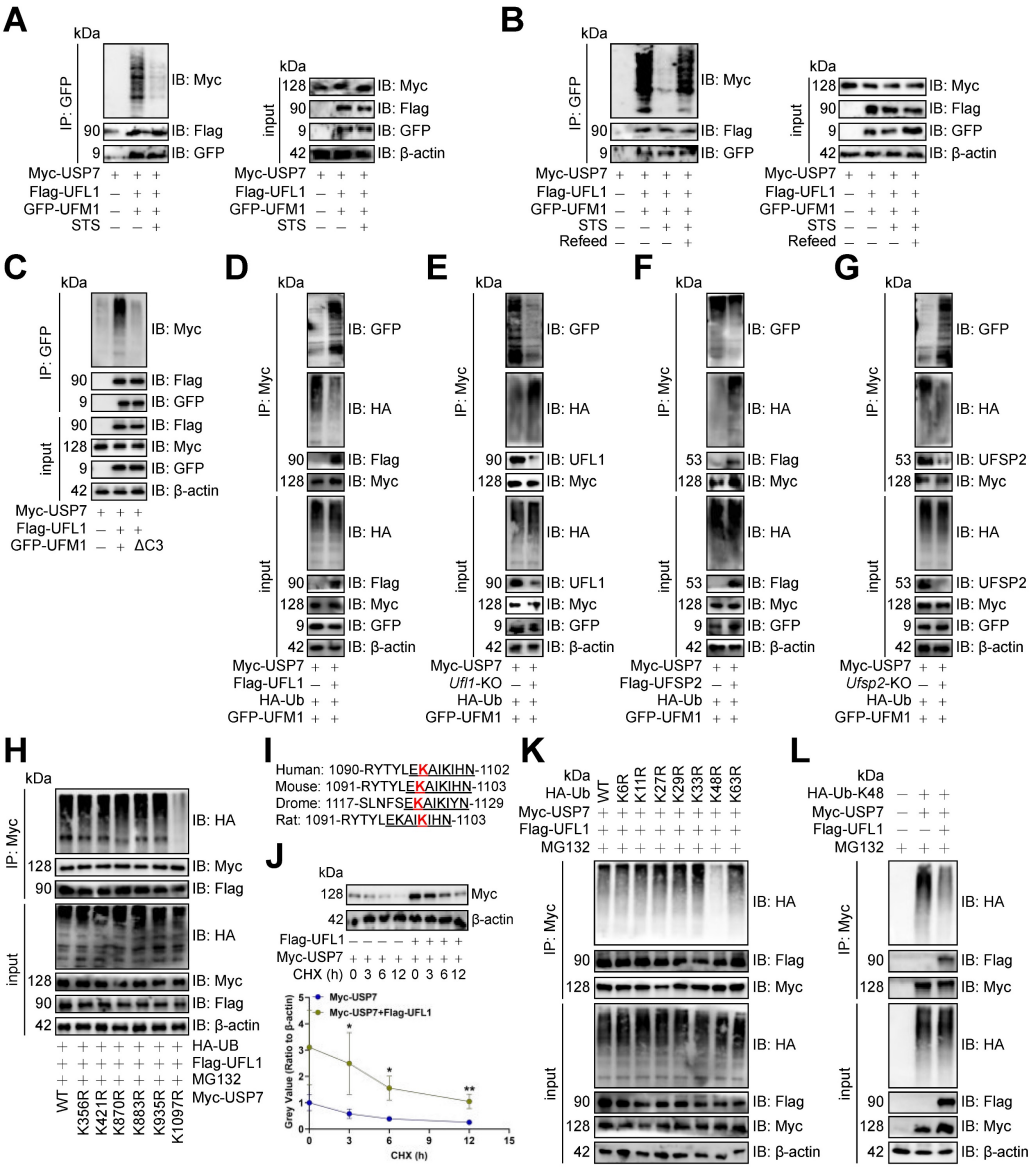
STS inhibited USP7 UFMylation thus increasing its ubiquitination.** (A-C)** Effects of short-term starvation **(A)**, refeed **(B)**, and deletion of the last three amino acid residues (83Gly-Ser-Cys85, ΔC3) of UFM1 **(C)** on USP7 UFMylation (*n* = 3). CD3^+^ T cells were co-transfected with Myc-labeled USP7, Flag-labeled UFL1, and GFP-labeled WT or deletion mutants of UFM1. **(D-G)** Effects of OE **(D, F)** or KO **(E, G)** of *Ufl1* **(D, E)** or *Ufsp2* **(F, G)** on USP7 UFMylation in CD3^+^ T cells (*n* = 3). **(H)** Polyubiquitination of six Myc-labeled USP7 lysine mutants in CD3^+^ T cells (*n* = 3). **(I)** Cross-species conservation of K1097 of USP7. Red indicated lysine site linked to ubiquitin. Underline indicated the same amino acid sequence. **(J)** CD3^+^ T cells with Myc-labeled USP7 were treated with Cycloheximide and USP7 protein expression was evaluated by immunoblotting (*n* = 3). **(K)** Ubiquitination chain analysis of USP7 in CD3^+^ T cells (*n* = 3). **(L)** Effects of UFL1 on K48-linked USP7 ubiquitination in CD3^+^ T cells (*n* = 3). **(J)** represented mean ± SD analyzed by unpaired *t* test. *P <0.05, **P <0.01. KO, knockout; OE, overexpression; WT, wild-type.

Since USP7 underwent UFMylation, we assessed STS's impact on it and found that STS inhibited USP7 UFMylation in CD3^+^ T cells (Figure 7A), whereas refeed enhanced it (Figure 7B). UFL1 is known to stabilize substrates via UFMylation 5, prompting us to investigate its effect on USP7. We demonstrated that UFL1 enhanced USP7 UFMylation in CD3^+^ T cells (Figure 7C). The inactive UFM1 form, achieved by removing the last three C-terminal amino acids (83Gly-Ser-Cys85, ΔC3) 46, effectively stopped USP7 UFMylation (Figure 7C). Furthermore, UFL1 enhanced USP7 UFMylation and reduced its ubiquitination in CD3^+^ T cells (Figure 7D, 7E), while UFSP2 decreased USP7 UFMylation and increased its ubiquitination (Figure 7F, 7G).

Furthermore, we examined USP7 ubiquitination by creating lysine (K)-arginine (R) mutants of potential sites from PhosphoSitePlus 47. Only K1097R stopped USP7 ubiquitination in CD3^+^ T cells (Figure 7H), and this site was highly conserved across species (Figure 7I). Moreover, *Ufl1*-OE enhanced USP7 protein stability in CD3^+^ T cells (Figure 7J). Finally, we mutated ubiquitin's K to R, finding K48R inhibited USP7 ubiquitination in CD3^+^ T cells (Figure 7K), while *Ufl1* reduced K48-linked its ubiquitination (Figure 7L).

### STS inhibited CD36 N-glycosylation and thus preventing USP7 UFMylation

**Figure 8:**
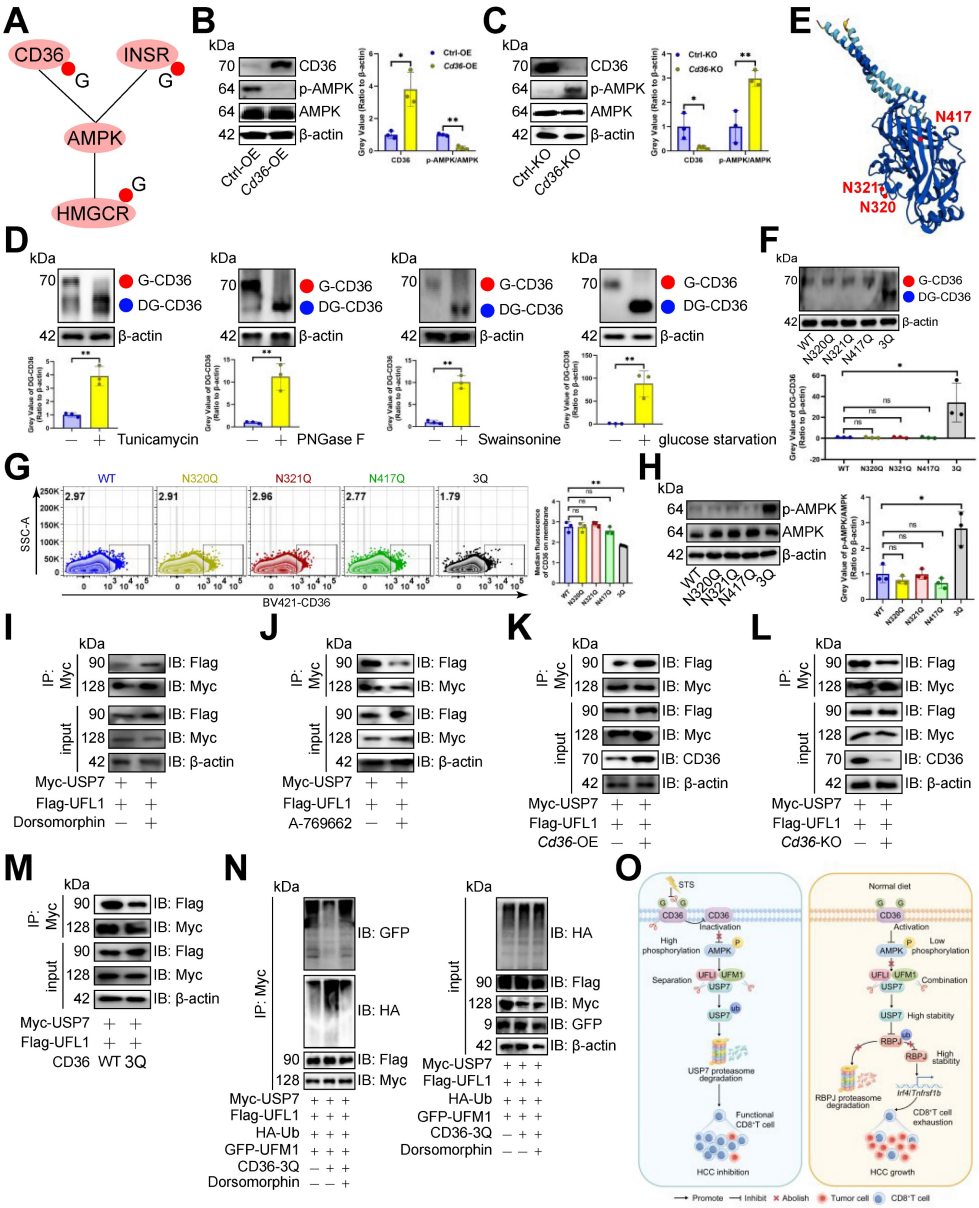
STS inhibited CD36 N-glycosylation and thus preventing USP7 UFMylation. **(A)** AMPK pathway-associated proteins that might undergo N-glycosylation. **(B, C)** Effects of OE **(B)** or KO **(C)** of *Cd36* on AMPK phosphorylation in CD3^+^ T cells (*n* = 3). **(D)** Effects of Tunicamycin, PNGase F, Swainsonine, and glucose starvation on CD36 N-glycosylation in CD3^+^ T cells (*n* = 3). **(E)** Protein structure and N-glycosylation site of CD36. **(F)** Effects of three possible N-glycosylated asparagine mutations to glutamine on CD36 expression in CD3^+^ T cells (*n* = 3). **(G)** Influence of mutations of three N-glycosylation sites on the membrane localization of CD36 on non-permeable CD3^+^ T cells (*n* = 3). **(H)** Effects of three N-glycosylation site mutations on AMPK phosphorylation in CD3^+^ T cells (*n* = 3). **(I-M)** Effects of AMPK inhibitor Dorsomophine **(I)**, AMPK agonist A-769662 **(J)**, *Cd36*-OE **(K)**, *Cd36*-KO **(L)**, or mutations of three N-glycosylation sites **(M)** on the binding of USP7 and UFL1 in CD3^+^ T cells (*n* = 3). **(N)** Influence of mutations of three N-glycosylation sites and Dorsomophine on USP7 UFMylation in CD3^+^ T cells (*n* = 3). **(O)** Mechanism diagram. **(B-D)** and **(F-H)** represented mean ± SD analyzed by unpaired *t* test. *P <0.05, **P <0.01. KO, knockout; OE, overexpression.

Since STS limits glucose intake 48 and reduces N-glycosylation 49, and energy inductor AMPK inhibits PD1 UFMylation 29, we proposed that STS regulates USP7 UFMylation to alleviate T cell exhaustion by limiting N-glycosylation of AMPK-associated proteins. Earlier data 50 revealed that CD36, INSR, and HMGCR, related to the AMPK pathway, might undergo N-glycosylation (Figure 8A). We found that *Cd36*-OE reduced p-AMPK/AMPK levels in CD3^+^ T cells (Figure 8B), while its deletion did the opposite (Figure 8C). However, HMGCR and INSR deletions didn't impact AMPK phosphorylation in CD3^+^ T cells (Figure S6A). Therefore, we hypothesized that CD36 is N-glycosylated and that this modification affects AMPK phosphorylation and USP7 UFMylation. We then used three N-glycosylation inhibitors Tunicamycin, PNGase F, and Swainsonine on CD3^+^ T cells, which reduced CD36's molecular weight in CD3^+^ T cells (Figure 8D), confirming that the higher molecular weight form was indeed N-glycosylated CD36. Additionally, glucose starvation maintained CD36's higher molecular weight (Figure 8D). We then investigated CD36 N-glycosylation sites and identified N320, N321, and N417 as potential sites through the earlier data 50, 51 (Figure 8E). Simultaneous mutations at three sites (3Q) appeared to be necessary to suppress CD36 N-glycosylation in CD3^+^ T cells (Figure 8F). Furthermore, we investigated how CD36 N-glycosylation affects its membrane localization and expression, finding that N-glycosylation inhibitors, glucose starvation, or 3Q of CD36 impaired CD36 membrane localization (Figure 8G, S6B, S6C) without altering its expression (Figure S6D, S6E) in CD3^+^ T cells. Furthermore, 3Q of CD36 increased p-AMPK/AMPK levels in CD3^+^ T cells (Figure 8H). Using the AMPK inhibitor Dorsomophine and the agonist A-769662 on CD3^+^ T cells, we observed that Dorsomophine increased USP7's binding to UFL1 (Figure 8I), whereas A-769662 decreased it (Figure 8J). This led us to hypothesize that CD36 N-glycosylation restricts USP7 UFMylation by blocking AMPK phosphorylation. As expected, *Cd36*-OE enhanced USP7's binding to UFL1 in CD3^+^ T cells (Figure 8K), whereas *Cd36*-KO or 3Q of CD36 prevented this interaction (Figure 8L, 8M). Finally, 3Q inhibited USP7 UFMylation and increased its ubiquitination in CD3^+^ T cells, a process blocked by Dorsomophine (Figure 8N).

### STS improved the immunotherapy efficacy of immunotherapy

Furthermore, given that STS had the potential to mitigate T cell exhaustion, we investigated its clinical significance and its synergistic value when combined with immune checkpoint inhibitors (ICIs) in conversion therapy. We constructed PDOX models and treated them with anti-PD1 and anti-CTLA4 antibodies combined with STS. We observed that both two ICIs and STS inhibited HCC growth (Figure S7A, S7B) and downregulated the expression of exhaustion genes PD1, TOX and TIGIT (Figure S7C, S7D). Meanwhile, STS enhanced two ICIs' effect on exhaustion gene expression suppression (Figure S7D). Both inhibitors also enhanced the therapeutic effect of STS (Figure S7D). Notably, while STS resulted in a 20% reduction of PD1 expression on CD4^+^ T cells, it achieved a 40% reduction on CD8^+^ T cells (Figure S7D). This observation aligned with the view suggesting that STS significantly impacted tumor-infiltrating CD8^+^ T cells rather than CD4^+^ T cells (Figure S1B). Finally, to evaluate the clinical relevance of USP7 or RBPJ expression and HCC immunotherapy response, we calculated the TIDE scores for the high and low expression groups of them. Our results showed that the low expression levels of these two reflected a more ideal treatment response rate (Figure S7E).

## Discussion

Energy metabolism regulates T cell differentiation, colonization, circulation, and response 52-54. Memory T cells in bone marrow are resting cells adapted for long-term survival with limited nutrients 52. Intermittent fasting influences T cell proliferation, with 8-week mild fasting boosting naive CD4^+^ T cell growth 53. A restrictive diet also reduces the expression of T cell exhaustion genes like PD1, TIM3, and KLRG1 54. This suggests that there is a complex interaction between STS and T cell function that needs further study. Our study showed that STS boosted T cell effector function, cytotoxicity, proliferation, and prevented exhaustion, thereby enhancing anti-tumor immunity. We connected N-glycosylation, UFMylation, and ubiquitination to metabolic/immune responses, highlighting that regulation of these PTMs was crucial for developing effective T cell functions under low glucose conditions.

UFMylation is crucial in immunotherapy 5, and targeting it could improve treatment outcomes 30. As a significant PTMs, UFMylation offers a promising approach for cancer therapy 30. In addition, the immune system is vital for controlling tumor growth, with T-cell-based therapies like ICI and CAR T-cell therapy being key cancer treatments 29. This study highlighted the importance of UFMylation in T cells, in regulating T cell activation and anti-tumor immunity. Previous studies indicated that eliminating PIRIN UFMylation induces ferroptosis of macrophages and boosts M1-type differentiation 55. Inhibiting PD1 UFMylation enhances T cell cytotoxicity 29. What's more, disrupting 14-3-3ε UFMylation activates antiviral responses and induces the expression of the interferon gene 56. In this study, we discovered that USP7 UFMylation prevented its ubiquitination, stabilized it, and aggravated T cell exhaustion. Therefore, the inhibition of UFMylation is indeed a key strategy for improving T cell function.

PTMs of transcription factors intricately interact with immune and inflammatory responses 57-59. Specifically, IRF3 up-regulates the expression of the ISGylation effector ISG15 while significantly downregulating thermogenic gene expression, a crucial mechanism through which inflammation suppresses adipose tissue thermogenesis 57. Furthermore, the ubiquitination pathway facilitates the activation of NF-κB signaling by promoting the phase separation of NEMO 58. Additionally, TRIM7/RNF90 enhances autophagy during infection by modulating the ubiquitination of ATG7 59. Our research underscored that the ubiquitination of the transcription factor RBPJ aggravated T cell exhaustion by stabilizing its expression. Furthermore, the interplay among PTMs is significant 60. This interaction is essential for accurate gene expression, maintenance of genome architecture, regulation of cell division, and the cellular response to DNA damage 60. Our research demonstrated that USP7 UFMylation suppressed its ubiquitination, subsequently inhibiting RBPJ ubiquitination. This finding underscored the intricate relationship between ubiquitination and UFMylation.

We acknowledged the potential limitations inherent in this study. Firstly, the PDOX model relies on the sensitivity of the patients used to construct the model to ICIs, which may not fully account for the TME in patients who exhibit insensitivity to these inhibitors. Considering this limitation, subsequent direct testing in human patients will be essential. Secondly, the dual KO of USP7 and RBPJ theoretically raises concerns regarding genotoxicity. Lastly, for clinical applications, it is imperative to recognize that if the elimination of USP7/RBPJ dual-edited cells is compromised, the resultant accumulation of a substantial number of highly proliferative and cytotoxic T cells could significantly elevate the risk of T cell lymphoma and cytokine storm. This issue can be mitigated through dose escalation studies and by incorporating STS to reduce the dosage. Considering these uncertainties, the preliminary clinical trials for this strategy should evaluate the application of gene therapy utilizing USP7/RBPJ dual-edited cells at comparatively low concentrations.

## Conclusions

Our study found that STS disrupted CD36 N-glycosylation, activated AMPK phosphorylation, reduced USP7 UFMylation, enhanced its ubiquitination, and destabilized USP7. This led to increased RBPJ ubiquitination and degradation, inhibiting the IRF4/TNFRSF1B axis and alleviating T cell exhaustion (Figure 8O). Our study highlighted the role of N-glycosylation, UFMylation, and ubiquitin in regulating T cell anti-tumor immunity.

## Supplementary Material

Supplementary figures.

## Abbreviations

MS: mass spectrometry
cKI: conditional knock-in
cKO: conditional knockout
CTR: C-terminal region
CyTOF: cytometry by time-of-flight
FDR: false discovery rate
HCC: hepatocellular carcinoma
ICI: immune checkpoint inhibitor
*i.p.*: intraperitoneally
LC-MS: liquid chromatography-mass spectrometry
NMDS: non-metric Multidimensional Scaling
NTR: N-terminal region
OE: overexpression
PDOX: patient-derived orthotopic xenograft
PTMs: protein translational modifications
RT: room temperature
STS: short-term starvation
TME: tumor microenvironment
WT: wild-type
