# Supplementary material for: Short-term starvation inhibits CD36 N-glycosylation and downregulates USP7 UFMylation to alleviate RBPJ-maintained T cell exhaustion in liver cancer
Source: Theranostics. 2025 Apr 28;15(12):5931–52. doi: 10.7150/thno.110567 (PMC12068301; doi:10.7150/thno.110567)
Supplement: Supplementary file 1 — Supplementary figures. [file thnov15p5931s1.pdf]

Supplementary Information:

Short-term starvation inhibits CD36 N-glycosylation and downregulates USP7 UFMylation to alleviate RBPJ-maintained T cell exhaustion in liver cancer

Banglun Pan, Siyan Chen, Hao Wu, Xiaoxia Zhang, Zhu Zhang, Dongjie Ye, Yuxin Yao, Yue Luo, Xinyu Zhang, Xiaoqian Wang, Nanhong Tang

Supplementary Figures and Legends

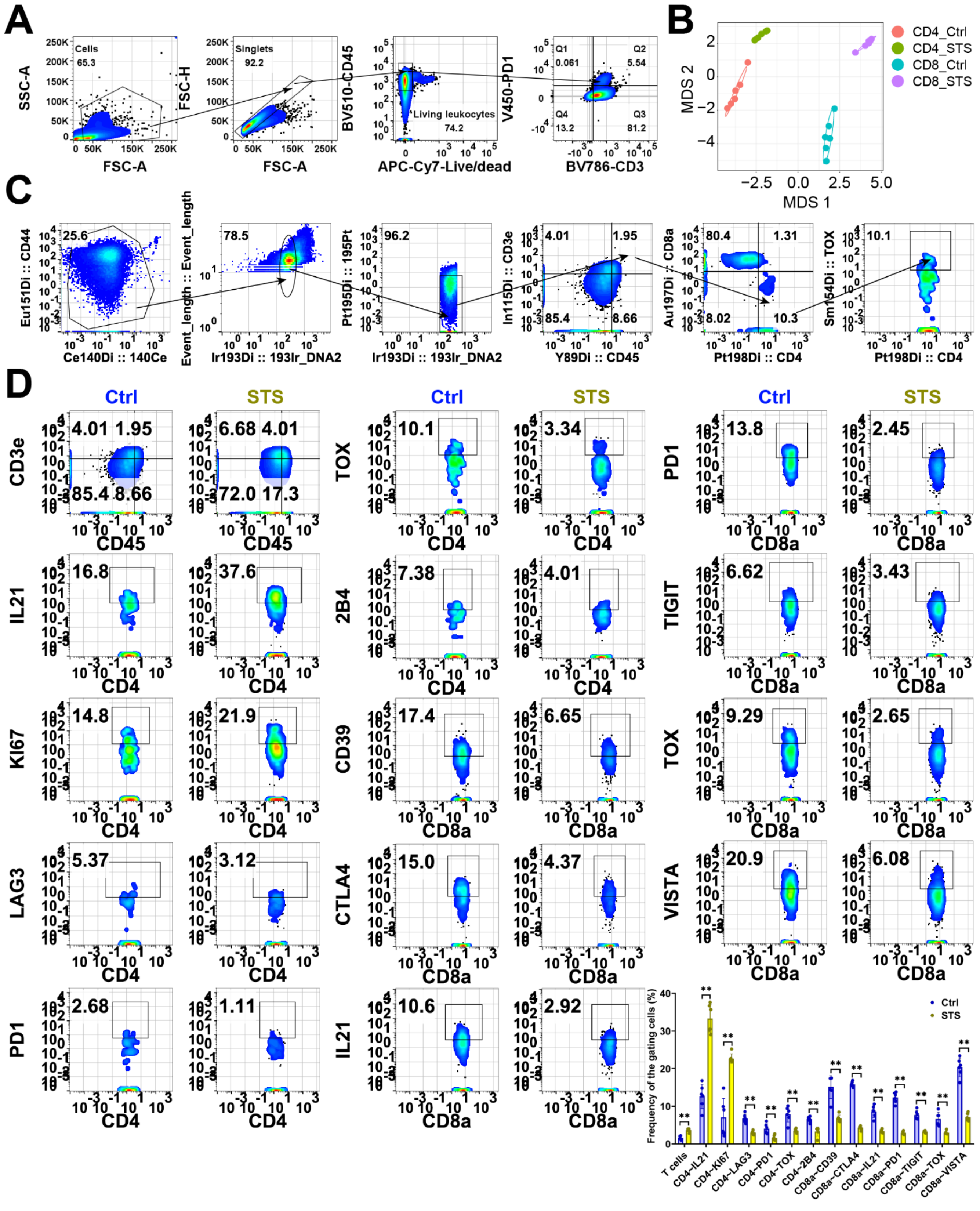

Figure S1. STS alleviated T cell exhaustion. (A) Flow cytometry gating logic showed T cell infiltration and its PD1 expression in primary cancer. (B) Non-metric Multidimensional Scaling analysis compared the similarity of $CD4^+$ and $CD8^+$ T cell characteristic antigen expression before and after STS ($n = 6$). (C, D) Mass cytometry revealed marker expression in $CD4^+$ and $CD8^+$ T cells. (C) Gating logic. (D) Representative plots and quantitative analysis ($n = 6$). (D) represented mean ± SD analyzed by unpaired *t* test. **P* <0.05, ***P* <0.01. STS, short-term starvation.

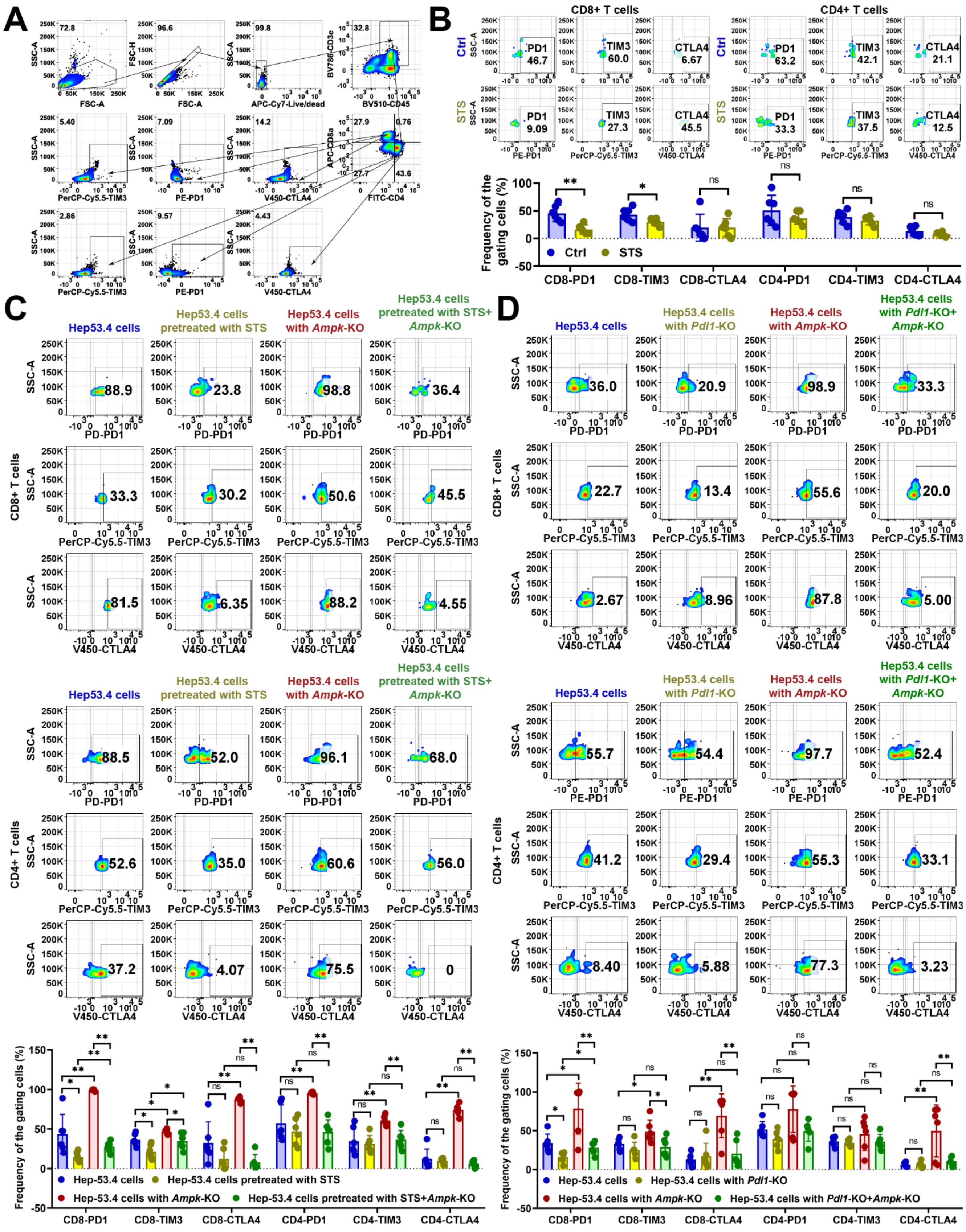
A
SSC-A
FSC-A
FSC-H
APC-Cy7-Live/dead
BV786-CD3e
BV510-CD45
APC-CD8a
FITC-CD4
PerCP-Cy5.5-TIM3
PE-PD1
V450-CTLA4
B
CD8+ T cells
CD4+ T cells
Ctrl
STS
PD1
TIM3
CTLA4
Frequency of the gating cells (%)
Ctrl
STS
CD8-PD1
CD8-TIM3
CD8-CTLA4
CD4-PD1
CD4-TIM3
CD4-CTLA4
C
Hep53.4 cells
Hep53.4 cells pretreated with STS
Hep53.4 cells with Ampk-KO
Hep53.4 cells pretreated with STS+ Ampk-KO
CD8+ T cells
CD4+ T cells
PD-PD1
PerCP-Cy5.5-TIM3
V450-CTLA4
Frequency of the gating cells (%)
Hep-53.4 cells
Hep-53.4 cells pretreated with STS
Hep-53.4 cells with Ampk-KO
Hep-53.4 cells pretreated with STS+Ampk-KO
D
Hep53.4 cells
Hep53.4 cells with Pdl1-KO
Hep53.4 cells with Ampk-KO
Hep53.4 cells with Pdl1-KO+ Ampk-KO
PE-PD1
Hep-53.4 cells
Hep-53.4 cells with Pdl1-KO
Hep-53.4 cells with Ampk-KO
Hep-53.4 cells with Pdl1-KO+Ampk-KO

Figure S2. STS prevented T cell exhaustion in vitro. (A) Gating logic. (B) Impact of STS on the expression of inhibitory receptors on $CD3^+$ T cells in vitro ($n = 6$). (C) Influence of STS pre-stimulated or *Ampk*-deficient Hep-53.4 cells on the expression of inhibitory receptors on $CD3^+$ T cells in vitro ($n = 6$). (D) Effect of Hep-53.4 cells with *Pdl1* or *Ampk* knockout on the expression of inhibitory receptors on $CD3^+$ T cells in vitro ($n = 6$). (B-D) represented mean ± SD analyzed by unpaired *t* test. **P* <0.05, ***P* <0.01. STS, short-term starvation.

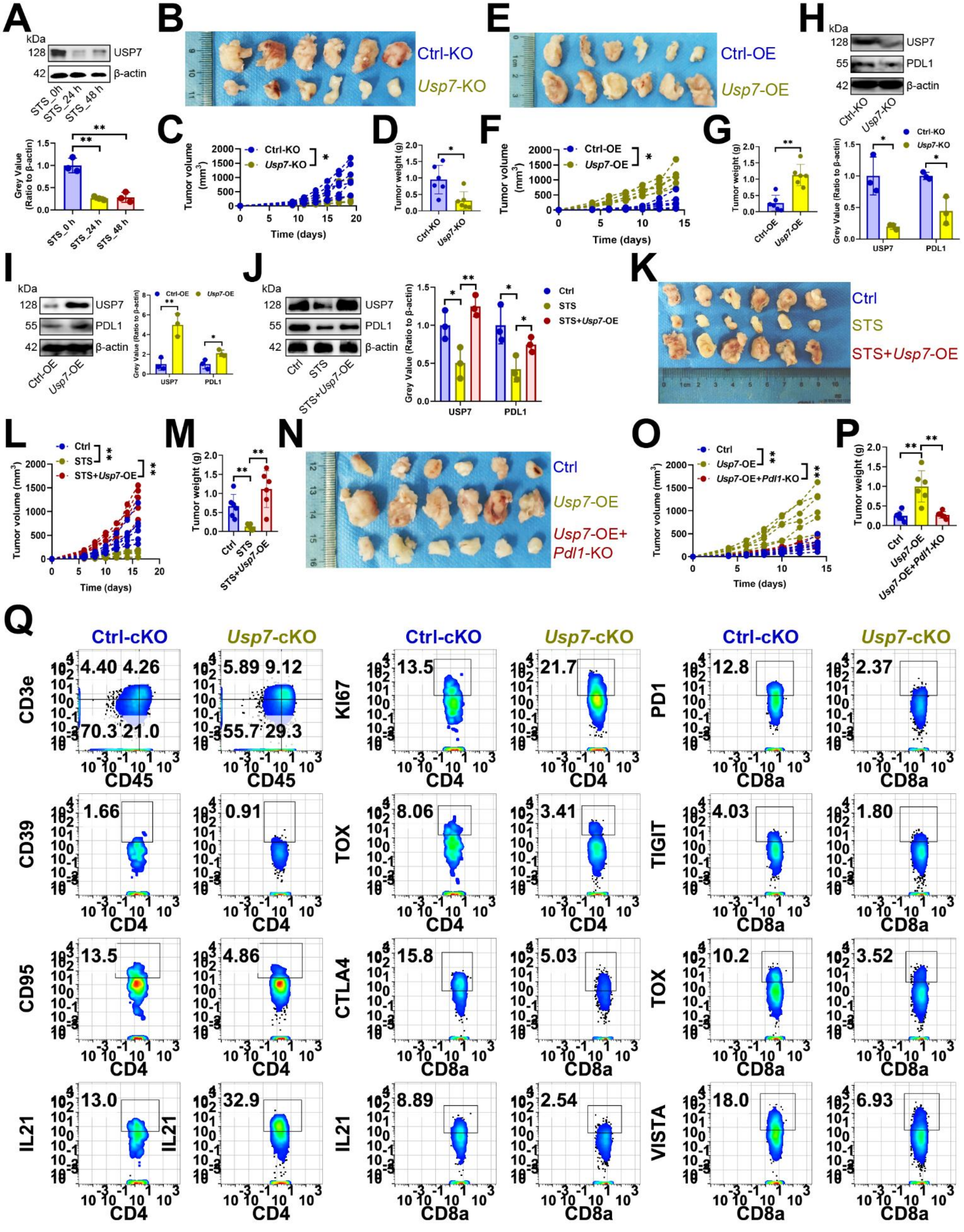

A
kDa
128
USP7
42
β-actin
STS_0h
STS_24 h
STS_48 h
Grey Value (Ratio to β-actin)
STS_0 h
STS_24 h
STS_48 h
B
Ctrl-KO
Usp7-KO
C
Ctrl-KO
Usp7-KO
Tumor volume (mm3)
Time (days)
D
Tumor weight (g)
Ctrl-KO
Usp7-KO
E
Ctrl-OE
Usp7-OE
F
Ctrl-OE
Usp7-OE
Tumor volume (mm3)
Time (days)
G
Tumor weight (g)
Ctrl-OE
Usp7-OE
H
kDa
128
USP7
55
PDL1
42
β-actin
Ctrl-KO
Usp7-KO
Grey Value (Ratio to β-actin)
USP7
PDL1
I
kDa
128
USP7
55
PDL1
42
β-actin
Ctrl-OE
Usp7-OE
Grey Value (Ratio to β-actin)
J
kDa
128
USP7
55
PDL1
42
β-actin
Ctrl
STS
STS+Usp7-OE
Grey Value (Ratio to β-actin)
K
Ctrl
STS
STS+Usp7-OE
L
Ctrl
STS
STS+Usp7-OE
Tumor volume (mm3)
Time (days)
M
Tumor weight (g)
Ctrl
STS
STS+Usp7-OE
N
Ctrl
Usp7-OE
Usp7-OE+
Pdl1-KO
O
Ctrl
Usp7-OE
Usp7-OE+Pdl1-KO
Tumor volume (mm3)
Time (days)
P
Tumor weight (g)
Ctrl
Usp7-OE
Usp7-OE+Pdl1-KO
Q
Ctrl-cKO
Usp7-cKO
Ctrl-cKO
Usp7-cKO
Ctrl-cKO
Usp7-cKO
CD3e
4.40 4.26
70.3 21.0
5.89 9.12
55.7 29.3
CD45
KI67
13.5
21.7
PD1
12.8
2.37
CD4
CD8a
CD39
1.66
0.91
TOX
8.06
3.41
TIGIT
4.03
1.80
CD95
13.5
4.86
CTLA4
15.8
5.03
TOX
10.2
3.52
IL21
13.0
32.9
IL21
8.89
2.54
VISTA
18.0
6.93

Figure S3. USP7 aggravated T cell exhaustion through promoting PDL1 expression in tumor cells. (A) Effects of STS on USP7 protein expression in Hep-53.4 cells (n = 3). (B-D) Effect of *Usp7*-KO in Hep-53.4 cells on subcutaneous tumor growth (n = 6). (B) Representative. (C) Growth curve. (D) Tumor weight. (E-G) Effect of *Usp7*-OE in Hep-53.4 cells on subcutaneous tumor growth (n = 6). (E) Representative. (F) Growth curve. (G) Tumor weight. (H) Effect of *Usp7*-KO on PDL1 protein expression in Hep-53.4 cells (n = 3). (I) Effect of *Usp7*-OE on PDL1 protein expression in Hep-53.4 cells (n = 3). (J) Effects of STS and *Usp7*-OE on PDL1 protein expression in Hep-53.4 cells (n = 3). (K-M) Effects of STS and *Usp7*-OE in Hep-53.4 cells on subcutaneous tumor growth (n = 6). (K) Representative. (L) Growth curve. (M) Tumor weight. (N-P) Effects of *Usp7*-OE and *Pdl1*-KO in Hep-53.4 cells on subcutaneous tumor growth (n = 6). (N) Representative. (O) Growth curve. (P) Tumor weight. (Q) Mass cytometry gating logic revealed marker expression in primary carcinoma-infiltrating $CD4^+$ and $CD8^+$ T cells. (A), (C), (D), (F-J), (L), (M), (O), and (P) represented mean ± SD analyzed by unpaired t test. *P <0.05, **P <0.01. KO, knockout; OE, overexpression; STS, short-term starvation.

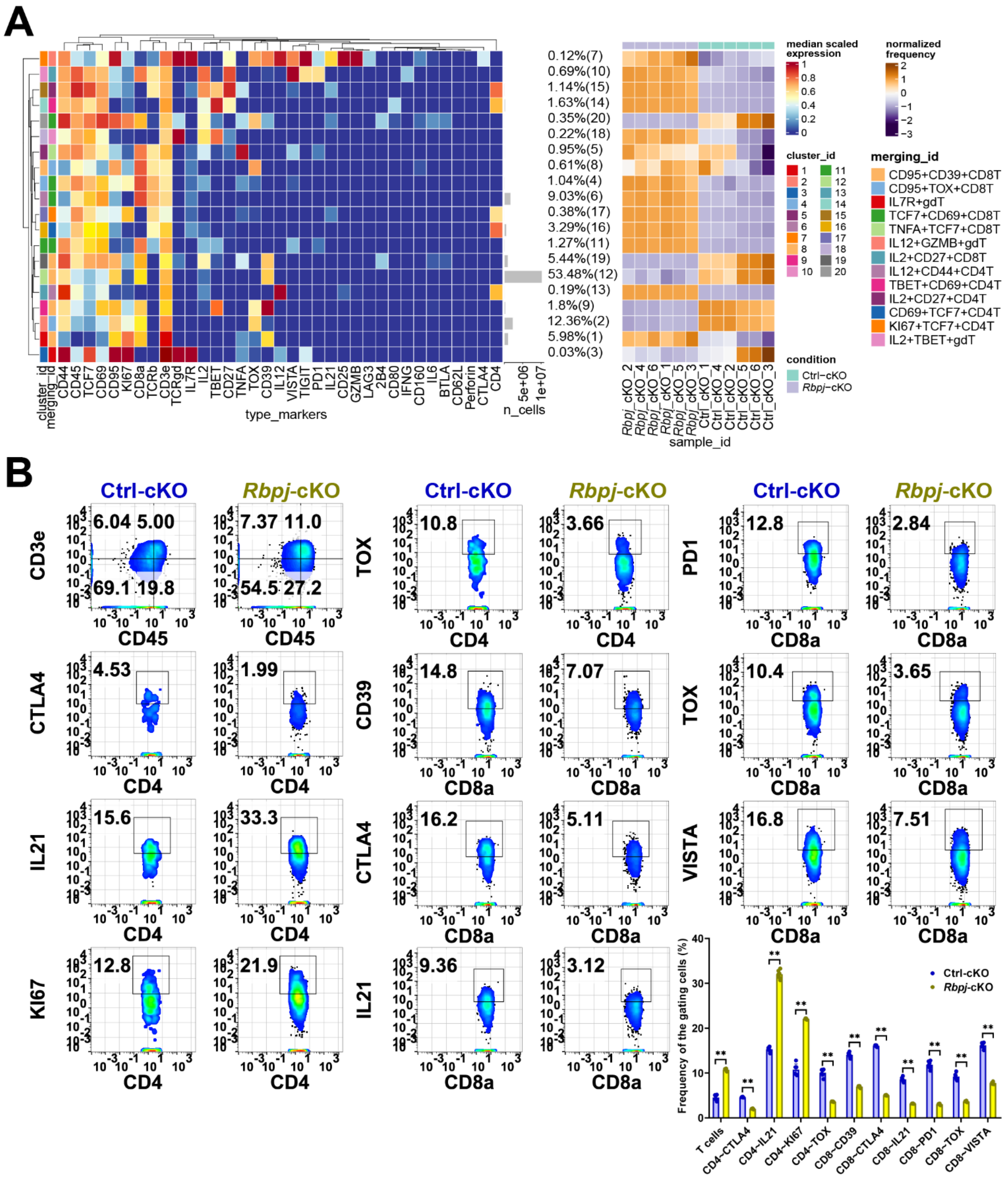

Figure S4. RBPJ aggravated T cell exhaustion. (A) Heatmap showed the median expression of the antigen used to generate self-organizing map (n = 6). (B) Mass cytometry revealed marker expression

in primary carcinoma-infiltrating $CD4^+$ and $CD8^+$ T cells ($n = 6$). (A) was analyzed by Euclidean Distance Clustering Algorithm, (B) represented mean ± SD analyzed by unpaired *t* test. $^{**}P < 0.01$.

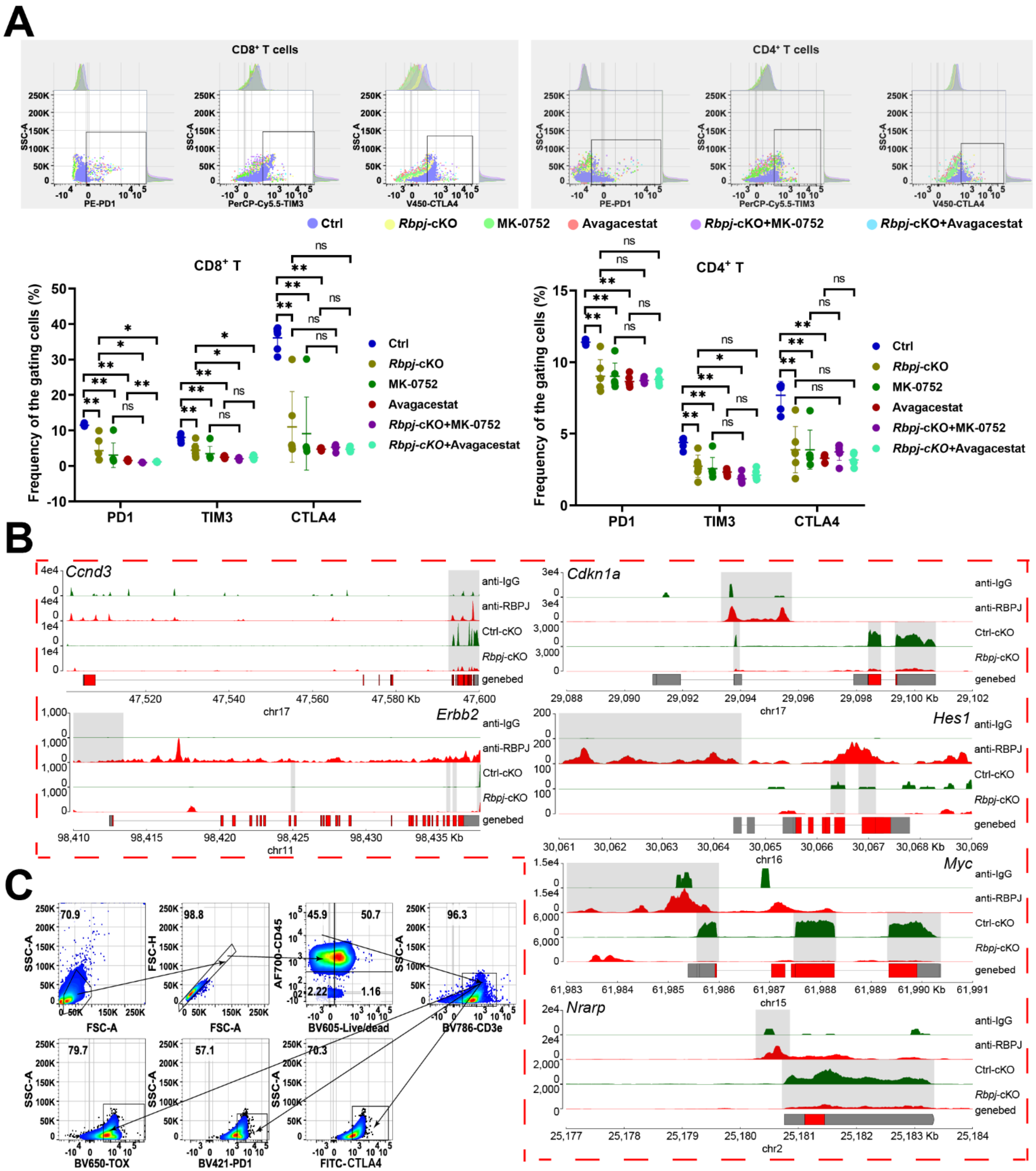

Figure S5. RBPJ aggravated T cell exhaustion not entirely dependent on Notch1. (A) Effects of *Rbpj*-cKO, γ-secretase inhibitor MK-0752, Notch1/γ-secretase inhibitor Avagacestat on the expression of

inhibitory receptors in primary carcinoma-infiltrating $CD4^+$ and $CD8^+$ T cells (n = 6). (B) Snapshot plots showed explicit transcription expression of Notch pathway-related genes and the enrichment signal of RBPJ on their promoters in $CD3^+$ T cells (n = 3). Gray indicated the differential signal. (C) Flow cytometry gating logic presented the expression of inhibitory receptors in $CD3^+$ T cells with *Rbpj*-cKO and *Irf4* or *Tnfrsf1b* overexpressed. (A) represented mean ± SD analyzed by unpaired *t* test. *P <0.05, **P <0.01. cKO, conditional knockout.

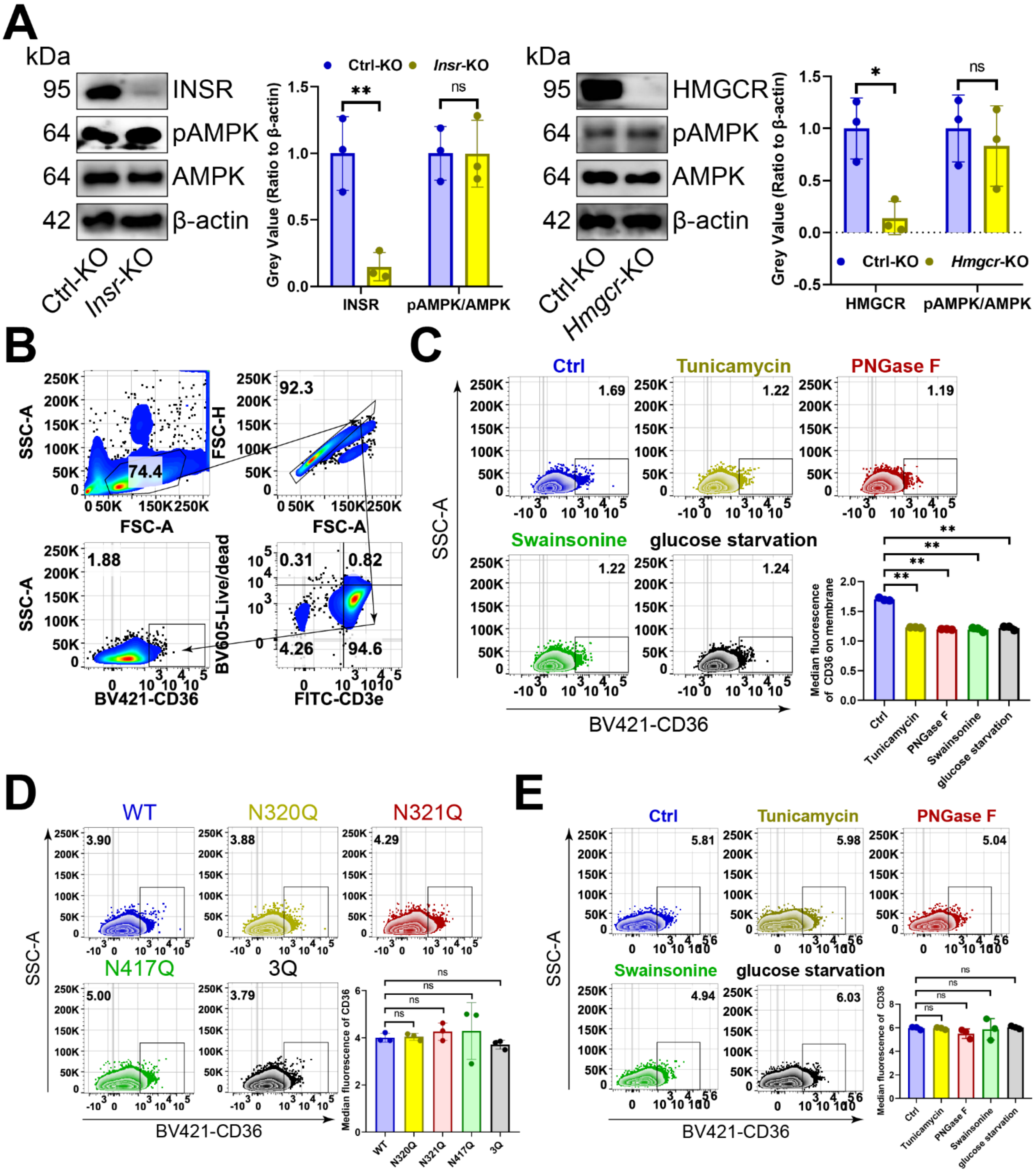

Figure S6. STS disrupted CD36 membrane localization rather than expression by inhibiting its N-glycosylation. (A) Effects of knockout of *Insr* or *Hmgcr* on AMPK phosphorylation in $CD3^+$ T cells (*n*

= 3). (B-E) Flow cytometry showed CD36 expression in primary carcinoma-infiltrating $CD3^+$ T cells. (B) Gating logic. (C) Influence of N-glycosylation inhibitors and glucose starvation on the membrane localization of CD36 on non-permeable $CD3^+$ T cells (n = 3). (D) Effect of mutations of three N-glycosylation sites on the expression of CD36 on permeable $CD3^+$ T cells (n = 3). (E) Impact of N-glycosylation inhibitors and glucose starvation in the expression of CD36 on permeable $CD3^+$ T cells (n = 3). (A, C-E) represented mean ± SD analyzed by unpaired *t* test. **P* <0.05, ** *P* <0.01. STS, short-term starvation.

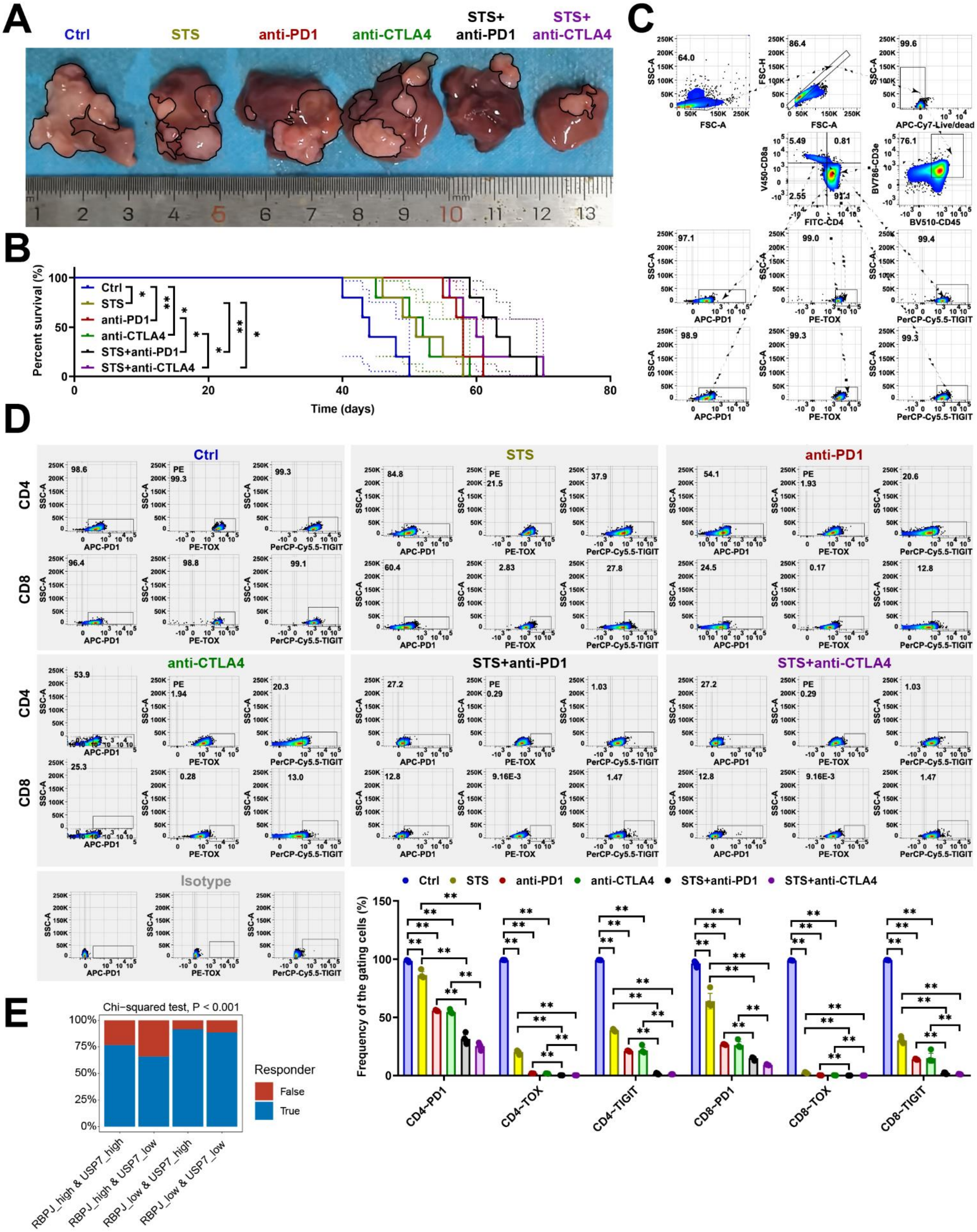
A
Ctrl
STS
anti-PD1
anti-CTLA4
STS+ anti-PD1
STS+ anti-CTLA4
B
Percent survival (%)
Time (days)
Ctrl
STS
anti-PD1
anti-CTLA4
STS+anti-PD1
STS+anti-CTLA4
C
SSC-A
FSC-A
FSC-H
APC-Cy7-Live/dead
V450-CD8a
FITC-CD4
BV786-CD3e
BV510-CD45
APC-PD1
PE-TOX
PerCP-Cy5.5-TIGIT
D
CD4
CD8
Ctrl
STS
anti-PD1
anti-CTLA4
STS+anti-PD1
STS+anti-CTLA4
Isotype
Frequency of the gating cells (%)
CD4−PD1
CD4−TOX
CD4−TIGIT
CD8−PD1
CD8−TOX
CD8−TIGIT
E
Chi−squared test, P < 0.001
Responder
False
True
RBPJ_high & USP7_high
RBPJ_high & USP7_low
RBPJ_low & USP7_high
RBPJ_low & USP7_low

Figure S7. STS improved the immunotherapy efficacy of immunotherapy. **(A, B)** Influence of two ICIs with STS on patient-derived orthotopic xenograft growth (n = 5). (A) Representative. (B) Survival curve. **(C, D)** Flow cytometry analysis of two ICIs and STS on PD1, TOX, and TIGIT expression in $CD4^+$ or $CD8^+$ T cells. (C) Gating logic. (D) Representative plots and quantitative analysis (n = 5). (E) TIDE scores demonstrated susceptibility of immunotherapy in groups with high and low USP7 or RBPJ expression (n = 371). (B) was analyzed by Log-rank test, (D) represented mean ± SD analyzed by unpaired t test. *P <0.05, **P <0.01. ICI, immune checkpoint inhibitor; STS, short-term starvation.
